# Comparative genome-wide analysis and evolutionary history of haemoglobin-processing and haem detoxification enzymes in malarial parasites

**DOI:** 10.1186/s12936-016-1097-9

**Published:** 2016-01-29

**Authors:** Patrath Ponsuwanna, Theerarat Kochakarn, Duangkamon Bunditvorapoom, Krittikorn Kümpornsin, Thomas D. Otto, Chase Ridenour, Kesinee Chotivanich, Prapon Wilairat, Nicholas J. White, Olivo Miotto, Thanat Chookajorn

**Affiliations:** Genomic and Evolutionary Medicine Unit, Centre of Excellence in Malaria, Faculty of Tropical Medicine, Mahidol University, Bangkok, Thailand; Department of Biochemistry, Faculty of Science, Mahidol University, Bangkok, Thailand; Division of Medical Genetics, Department of Medicine, Faculty of Medicine Siriraj Hospital, Bangkok, Thailand; Division of Molecular Genetics, Department of Research and Development, Faculty of Medicine Siriraj Hospital, Mahidol University, Bangkok, Thailand; Parasite Genomics, Wellcome Trust Sanger Institute, Wellcome Trust Genome Campus, Cambridge, UK; Mahidol-Oxford Tropical Medicine Research Unit, Faculty of Tropical Medicine, Mahidol University, Bangkok, Thailand; Department of Clinical Tropical Medicine, Faculty of Tropical Medicine, Mahidol University, Bangkok, Thailand; Centre for Tropical Medicine, Nuffield Department of Medicine, University of Oxford, Oxford, UK; Wellcome Trust Sanger Institute, Hinxton, UK; Medical Research Council (MRC) Centre for Genomics and Global Health, University of Oxford, Oxford, UK

## Abstract

**Background:**

Malaria parasites have evolved a series of intricate mechanisms to survive and propagate within host red blood cells. Intra-erythrocytic parasitism requires these organisms to digest haemoglobin and detoxify iron-bound haem. These tasks are executed by haemoglobin-specific proteases and haem biocrystallization factors that are components of a large multi-subunit complex. Since haemoglobin processing machineries are functionally and genetically linked to the modes of action and resistance mechanisms of several anti-malarial drugs, an understanding of their evolutionary history is important for drug development and drug resistance prevention.

**Methods:**

Maximum likelihood trees of genetic repertoires encoding haemoglobin processing machineries within *Plasmodium* species, and with the representatives of Apicomplexan species with various host tropisms, were created. Genetic variants were mapped onto existing three-dimensional structures. Genome-wide single nucleotide polymorphism data were used to analyse the selective pressure and the effect of these mutations at the structural level.

**Results:**

Recent expansions in the falcipain and plasmepsin repertoires are unique to human malaria parasites especially in the *Plasmodium falciparum* and *P. reichenowi* lineage. Expansion of haemoglobin-specific plasmepsins occurred after the separation event of *Plasmodium* species, but the other members of the plasmepsin family were evolutionarily conserved with one copy for each sub-group in every Apicomplexan species. Haemoglobin-specific falcipains are separated from invasion-related falcipain, and their expansions within one specific locus arose independently in both *P. falciparum* and *P. vivax* lineages. Gene conversion between *P. falciparum* falcipain 2A and 2B was observed in artemisinin-resistant strains. Comparison between the numbers of non-synonymous and synonymous mutations suggests a strong selective pressure at falcipain and plasmepsin genes. The locations of amino acid changes from non-synonymous mutations mapped onto protein structures revealed clusters of amino acid residues in close proximity or near the active sites of proteases.

**Conclusion:**

A high degree of polymorphism at the haemoglobin processing genes implicates an imposition of selective pressure. The identification in recent years of functional redundancy of haemoglobin-specific proteases makes them less appealing as potential drug targets, but their expansions, especially in the human malaria parasite lineages, unequivocally point toward their functional significance during the independent and repetitive adaptation events in malaria parasite evolutionary history.

**Electronic supplementary material:**

The online version of this article (doi:10.1186/s12936-016-1097-9) contains supplementary material, which is available to authorized users.

## Background

**Fig. 1:**
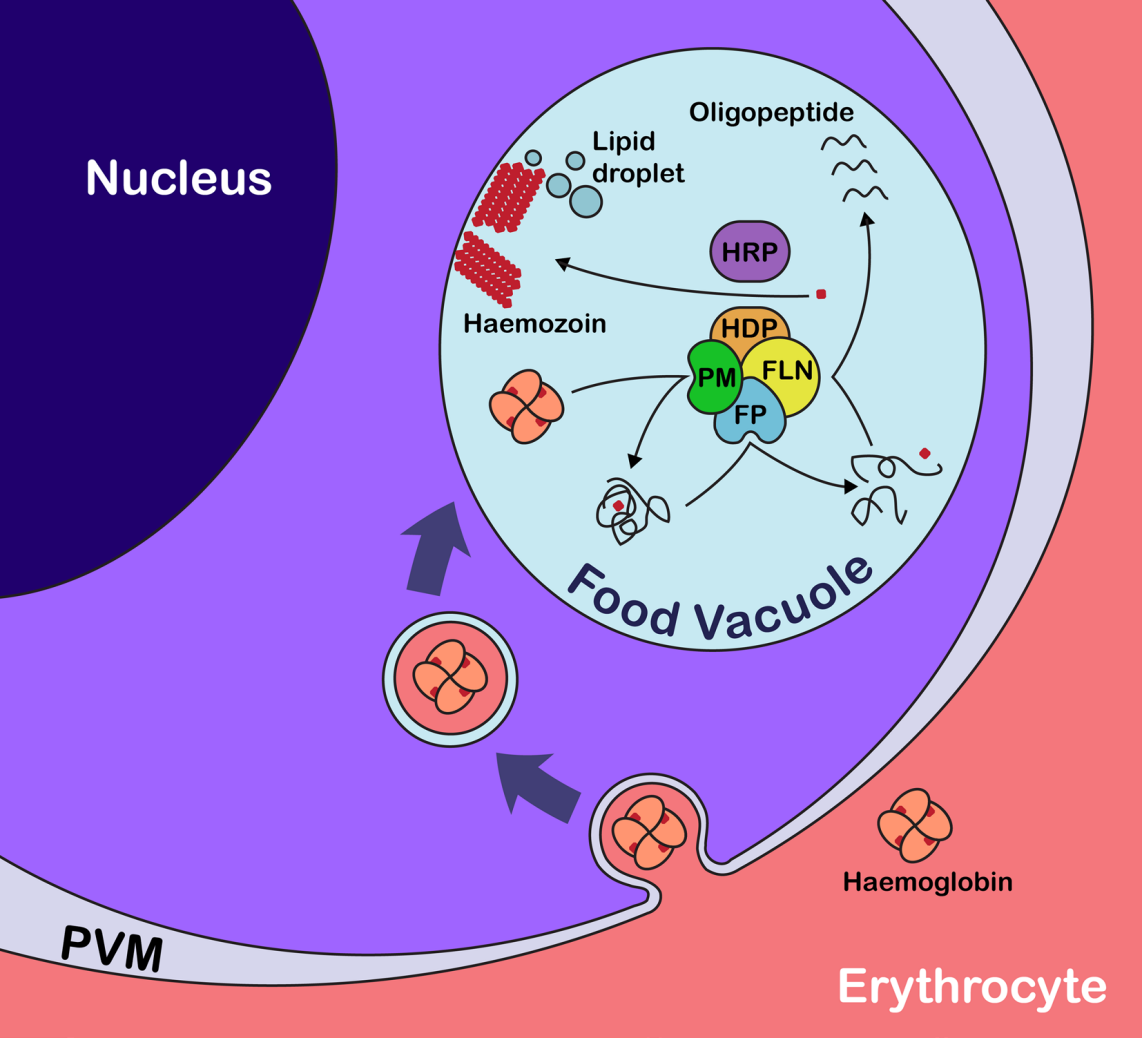
Diagram depicting haemoglobin degradation and haemozoin synthesis in *Plasmodium falciparum.* Haemoglobin is taken up into the parasite via cytostome, passing through parasite membrane and parasitophorous vacuole membrane (PVM). Double-membrane vesicle containing red blood cell content as observed in a cross-section delivers Hb into the food vacuole [50]. A series of proteases in the plasmepsin (PM), falcipain (FP) and falcilysin (FLN) family digest Hb into either free amino acid or short peptide. Haem from digested Hb is packed into polymerized crystal called haemozoin with help from haem detoxification protein (HDP) and histidine-rich protein (HRP). Lipid biomolecules from either food vacuole membrane or droplet are suggested to promote haemozoin synthesis probably by seeding and directing crystal growth [51]

Malaria parasites belong to the *Plasmodium* genus with four established human malaria species, namely, *Plasmodium falciparum*, *P.malariae*, *P.ovale,* and *P.vivax* [1]. Several *Plasmodium* parasites also infect non-human hosts, such as rodent (*P.berghei* and *P.chabaudi*) and chimpanzee (*P.reichenowi*) [2]. *Plasmodium* spp. belong to the Apicomplexa phylum and contain apicoplast, a plastid-like organelle that is an indicator of close evolutionary relationship between the ancestors of plants and apicomplexan species [3]. Several members of the Apicomplexa phylum are pathogens of human and veterinary diseases with the capability to infect a broad range of cell types. Malaria parasites are evolutionarily equipped with intricate machineries to degrade host haemoglobin (Hb) during their intra-erythrocytic stages of development. With an estimated Hb concentration of 5 mM, the red blood cell is an ideal host cell for supply of amino acid nutrient [4]. Nevertheless, Hb-rich environment could be a potentially hostile milieu for malaria parasites owing to the iron-containing haem released from the digested protein. *Plasmodium* employs a series of proteases for digesting globin and, lacking haem oxygenase within the acidic digestive vacuole, releases free haem molecules that form dimers linked together via H-bonds between the carboxyl side chains of the protoporphyrin rings [5]. This arrangement allows the formation of a crystal-like pigment (known as haemozoin) and keeps haem iron and free haem from causing oxidative and membrane damage (Fig. 1) [6]. It is worth tracing the evolutionary pathway by which a group of single-celled protozoa has achieved this remarkable feat. This issue is also of clinical importance in view of the number of anti-malarials that act by interfering with this Hb processing apparatus [7, 8]. Changes in genes encoding these proteins are known to alter anti-malarial sensitivity [9, 10]. Identifying the origin of malaria parasite Hb processing machinery and the effect of selective pressure on its evolution might help reveal *Plasmodium* variants specific to anti-malarial resistance.

In order for *Plasmodium* to evolutionarily become a parasite of red blood cells, it has to gain two functions, namely, Hb degradation and haem detoxification. The cellular machinery required for executing these two tasks has been biochemically and functionally characterized in *P*. *falciparum* to occur within a single protein complex (Fig. 1) [11]. The major component of the complex is haem detoxification protein (HDP), which catalyzes haem biocrystallization by tethering haem molecules together [12]. Three families of proteases, namely, cysteine protease (falcipain 2A and falcipain 2B), aspartic protease (plasmepsin II, III and IV) and metalloprotease (falcilysin) are associated with the complex [11]. These proteases have been functionally shown to target Hb [6]. The diversity and redundancy in Hb-targeted proteases indicate that the process of Hb degradation is vital for malaria survival as the evolutionary process has generated an array of proteases to accomplish the task. These proteases target different Hb parts and act sequentially to degrade globin chains [6].

Genetic alterations of Hb processing genes are linked to loss in anti-malarial drug sensitivity. For instance, knock-out of falcipain 2A reduces artemisinin sensitivity [13]. Interestingly, a mutation in falcipain 2A also arose during long-term selection of artemisinin resistance [14]. Both artemisinin and chloroquine inhibit HDP complex function, but with different modes of action [10, 11]. In addition, quinolone anti-malarials also affect Hb degradation and haem detoxification [7]. The selective pressure from such drugs is likely to alter the course of parasite evolution through changes in genes encoding members of the HDP complex, especially in ‘hotspot’ areas of drug resistance in Southeast Asia, where there is well-established evidence for loss in artemisinin sensitivity [15].

Here, the evolution history of *Plasmodium* protease families known to target Hb, including members of the HDP complex, was traced. Variations of *P*. *falciparum* HDP complex genes obtained from subjects in hotspot areas, including those from emerging artemisinin-resistant isolates, were analysed and mapped onto protein structures. Genes under selective pressure and mutations at key positions are described.

## Methods

### Phylogenetic analysis

Amino acid sequences of falcipains, plasmepsins, falcilysin, and HDP from *P. falciparum* strain 3D7 were retrieved from NCBI Protein database (Additional file 1) and SNP data of *P. falciparum* candidate genes were obtained from MalariaGEN [16]. Sequences from *P. falciparum* were used as a BLAST query against non-redundant protein sequence database (nr). BLASTp parameters were set as follows: scoring matrix, BLOSUM62; gap penalty, 11; and, gap extension penalty, 1. BLASTp hits that fulfilled the following criteria were selected as candidates for further analysis: (1) E-value <10^−6^; (2) alignment score >80; and, (3) BLAST alignment covering the functional domain. Homologue candidates in other *Plasmodium* spp. (*P. reichenowi*, *P. vivax*, *P.knowlesi*, *P. berghei*, and *P.yoelii*) and other protozoa (*Babesia bovis*, *Babesia microti*, *Eimeria tenella*, *Toxoplasma gondii*, *Theileria parva*, and *Theileria annulata*) were retrieved from NCBI database. The DNA sequences encoding falcipain 2A and falcipain 2B from *P. reichenowi* PrCDC were obtained by de novo assembly [17]. Sequences of interest were aligned using MUSCLE [18, 19]. Neighbour-joining (NJ) tree and 1000 bootstrap replicates were constructed using ClustalX version 2.0 [20]. The best-fit protein evolutionary model was determined using ProtTest [21]. Unrooted maximum likelihood (ML) tree was estimated using RAxML software [22, 23] and constructed using Dendroscope version 3.2.10 [24].

### Nested-PCR and sequence analysis of falcipain genes

In nested-PCR amplification of falcipain 2A and falcipain 2B genes, first round PCR was performed in a 25-µl reaction containing 1X HF buffer (Thermo Scientific), 1.5 mM MgCl_2_ for falcipain 2A or 5 mM MgCl_2_ for falcipain 2B, 0.5 pmol of dNTPs, 10 pmol of each primer (Additional file 2), 25 ng of parasite DNA, and 0.2 U Phusion DNA polymerase (Thermo Scientific). PCR was performed in an Eppendorf Mastercycler Pro under the following conditions: 98 °C for 30 s; 35 cycles of 98 °C for 10 s, 55 °C for 30 s, and 72 °C (for falcipain 2A) or 68 °C (for falcipain 2B) for 135 s; and, 72 °C for 5 min. Second-round PCR was carried out as described above except that 1 µl of 10^−4^ dilution of first-round PCR solution, second-round PCR primers (Additional file 2) and the extension step of 72 °C for 45 s were used. The nested-PCR amplicons were sequenced in both directions in ABI3130XL DNA sequencer (Applied Biosystems).

### Homology modelling and structural analysis

Structural data of plasmepsin I (accession no 3QS1), plasmepsin II (accession no 1XDH), HAP (accession no 3FNT), falcipain 2A (accession no 1YVB), falcipain 3 (accession no 3BPM), and falcilysin (accession no 3S5M) were retrieved from RCSB Protein Data Bank. Homology model of falcipain 2B was constructed using SWISS-MODEL with falcipain 2A (accession no 1YVB) as a template, and the model was subsequently refined using AMBER03 force field in GROMACS and checked for Ramachandran outlier by RAMPAGE [25, 26]. Structural models were visualized by PyMOL.

## Results

### Evolution of *Plasmodium* Hb processing genes

Members of two *Plasmodium* protease families, falcipain and plasmepsin, have been functionally characterized as being Hb-specific [6]. Falcipain 2A, falcipain 2B, plasmepsin II, plasmepsin III, plasmepsin IV, and falcilysin are associated with the HDP complex (Fig. 1) [11], but both protease families also have members that function in pathways not related to Hb processing, and some are not expressed during the intra-erythrocytic stages [27].

### Plasmepsin family

**Fig. 2:**
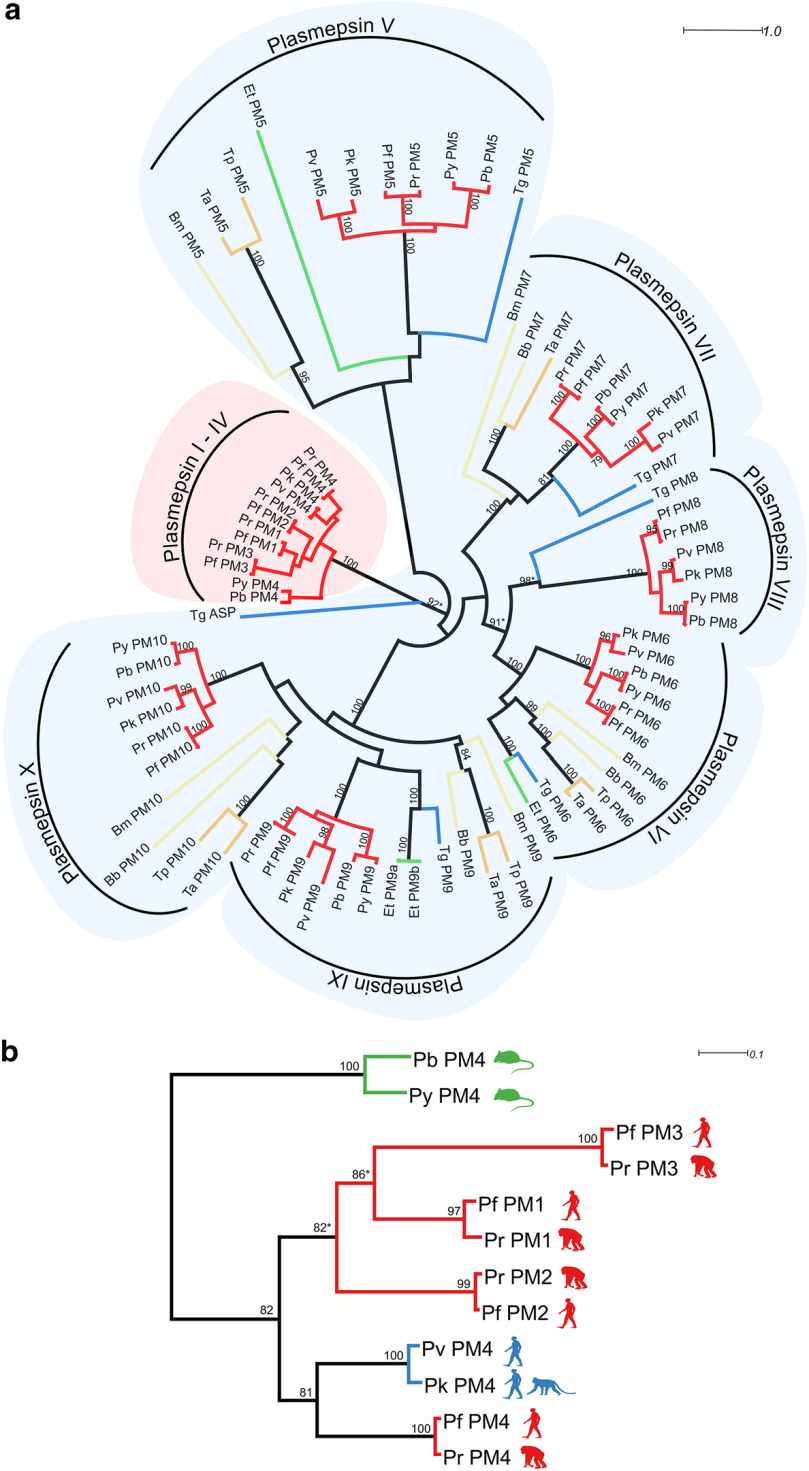
Haemoglobin-specific plasmepsin expansion in *Plasmodium falciparum.* **a** ML phylogenetic tree of plasmepsin. ML support value above 75 % based on 100 replicates bootstrap is shown on the branch except plasmepsin I-IV clade. *Plasmodium* species are grouped with the homologues from other members of Apicomplexa with one gene from each species. The tree shows separation of *P. falciparum* food vacuole plasmepsins (*pink area*) from plasmepsins localized in other cellular compartments or with different functions (*light blue area*). Sequences shown here were given code name according to their phylogenetic association with *P. falciparum* plasmepsin (see Additional file 1). *Red branch*: *Pf* *P. falciparum,* *Pr* *P. reichenowi,* *Pv* *P. vivax,* *Pk* *P. knowlesi,* *Pb* *P. berghei,* *Py* *P. yoelii,* *Yellow branch*: *Bb* *B. bovis,* *Bm* *B. microti,* *Orange branch*: *Tp* *T. parva,* *Ta* *T. annulata,* *Green branch*: *Et* *E. tenella,* *Blue branch*: *Tg* *T. gondii*. **b** ML phylogenetic tree specific to *Plasmodium* plasmepsins shows the expansion of food vacuole plasmepsins in the *P. falciparum*/*P. reichenowi* lineage (*red branch*). *P. vivax*/*P. knowlesi* (*blue branch*) and *P. berghei*/*P. yoelii* (*green branch*) have only one plasmepsin gene in this clade. ML support value above 75 % based on 100 replicates bootstrap is shown on the branch. *Star* indicates that the node has <75 % bootstrap support based on 1000 replicates for NJ tree

The phylogenetic tree of plasmepsin family reveals an expansion of food vacuole-specific Hb-targeting enzymes, namely, plasmepsin I–IV (Fig. 2). This clade contains a cluster of four food vacuole plasmepsins in *P. falciparum* and *P. reichenowi* while other *Plasmodium* spp., i.e., *P. vivax*, *P. knowlesi*, *P. berghei*, and *P. yoelii*, have one plasmepsin member in the clade (Fig. 2). The remaining members of the plasmepsin family, plasmepsin V-X, from every *Plasmodium* sp. can be grouped with homologues from other species of Apicomplexa with one member from each species (Fig. 2).

### Falcipain family

**Fig. 3:**
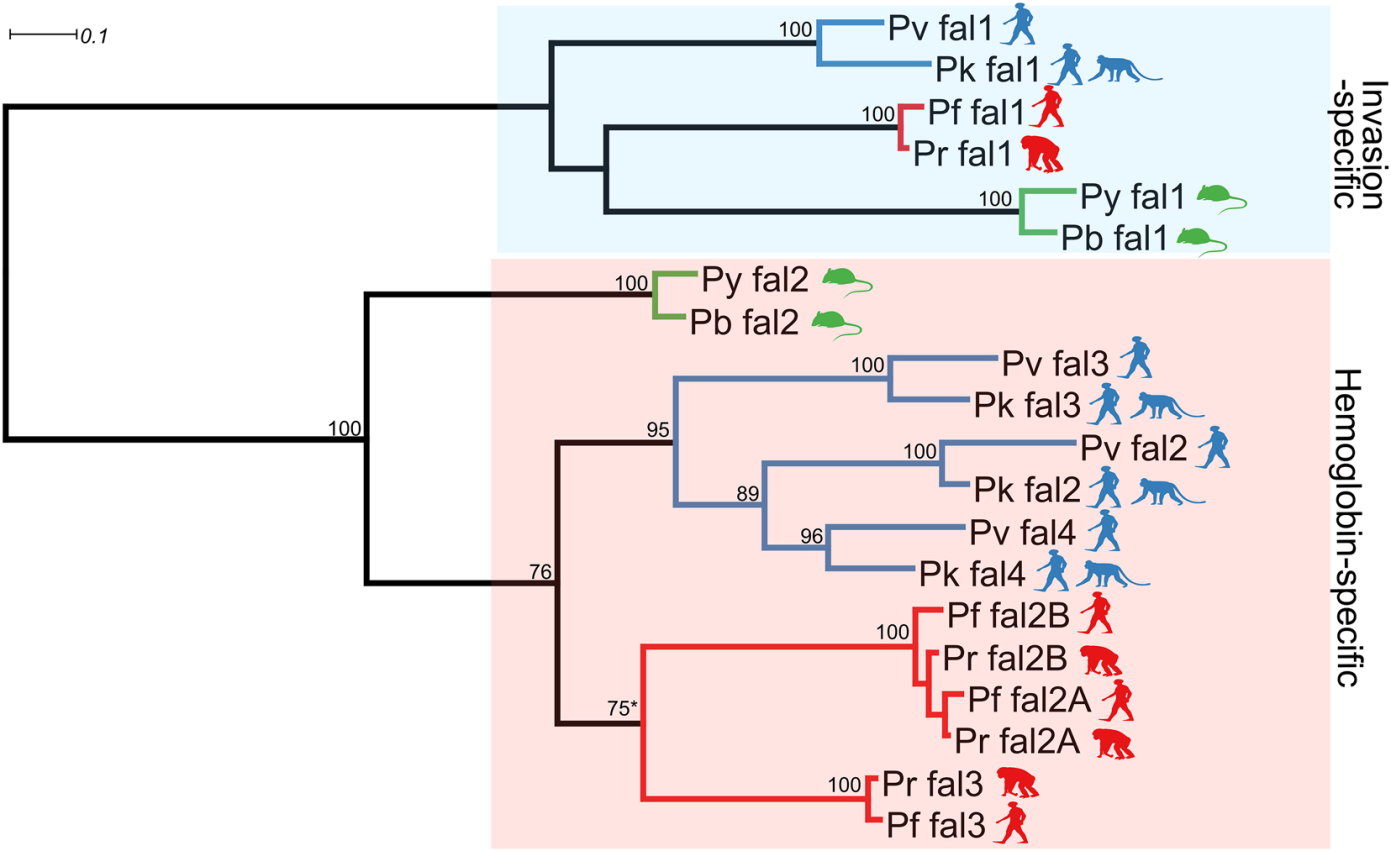
ML phylogenetic tree of *Plasmodium* falcipains. *Plasmodium* falcipains form two separated clades of Hb-specific falcipains (*pink area*) and invasion-specific falcipains (*light blue area*). The expansion of Hb-specific falcipains in human/primate *Plasmodium* occurred separately in *P. falciparum*/*P. reichenowi* lineage (*red branch line*) and *P. vivax*/*P. knowlesi* lineage (*blue branch line*). Falcipain expansion is not evident in *P. yoelii*/*P. berghei* lineage (*green branch line*). Numeric value shown on the branch indicates ML support value above 75 % based on 100 replicates bootstrap. NJ phylogenetic tree was also inferred. Bootstrap value for NJ tree was calculated from 1000 tree replicates. Both NJ and ML trees have comparable tree topology (data not shown) with high bootstrap support. Only ML tree is shown here. *Star* indicates that the node has <75 % NJ bootstrap support. Sequences shown here were given code name (see Additional file 1)

**Fig. 4:**
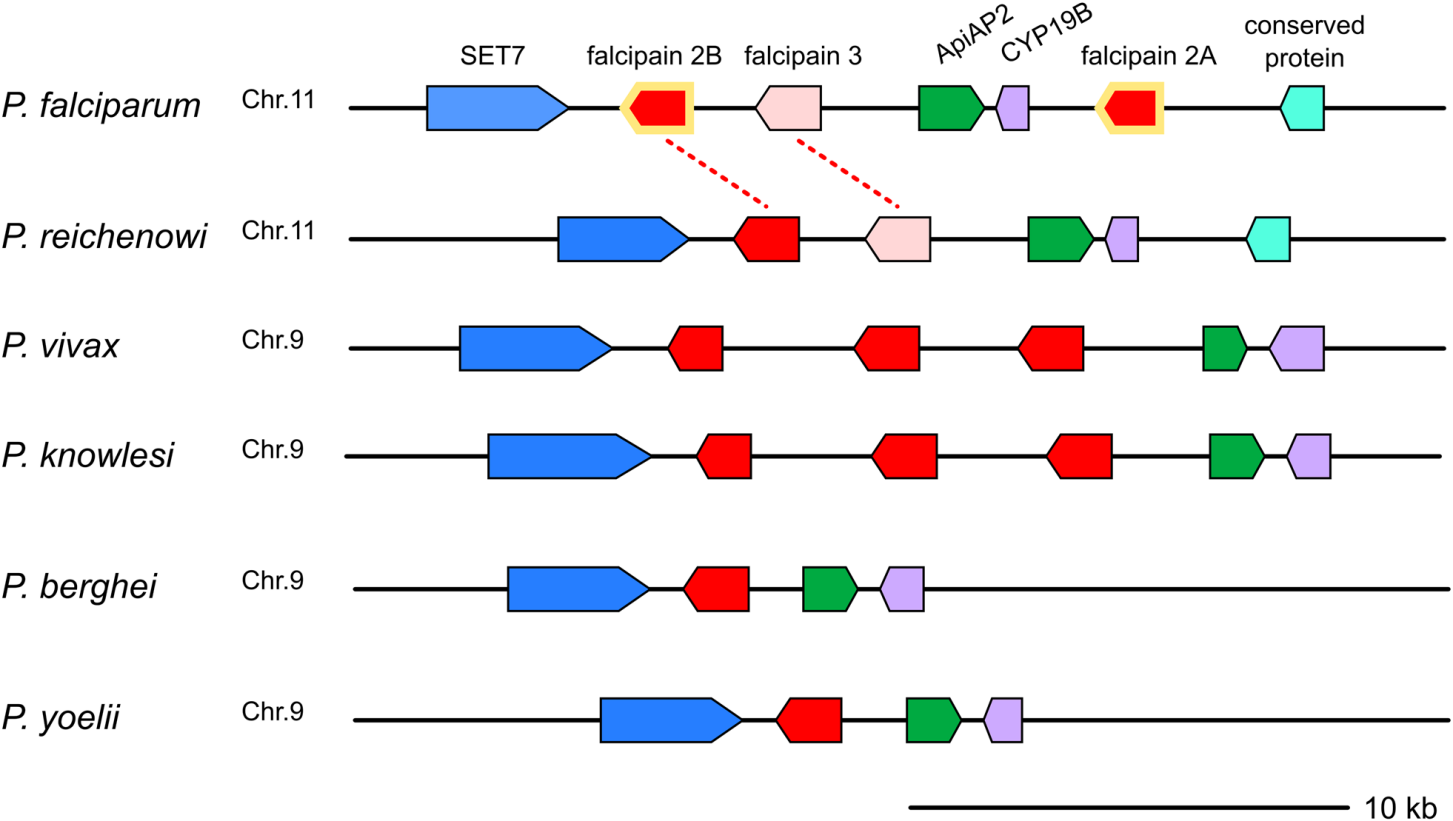
Food-vacuole falcipain loci in *Plasmodium.* Haemoglobin-specific falcipains (shown in *red and pink*) are located in close proximity as a falcipain locus. The genes surrounding the falcipain locus are conserved among *Plasmodium* species. Falcipain 2A of *P. reichenowi* is not shown here. *P. falciparum* falcipain 2A and falcipain 2B (*yellow border*) are almost identical to one another. *Red dashed lines* indicate falcipain 2B and falcipain 3 homologues in *P. reichenowi*. The *scaled line* length is approximately 10 kb

Expansion of Hb-specific proteases in this family is less distinct. Even though *Plasmodium* falcipains are clustered together, Hb-specific falcipains 2 and 3 are separated from invasion-specific falcipain 1 (Fig. 3 and Additional file 3: Figure S1) [28, 29]. *Plasmodium berghei* and *P*. *yoelii* have two falcipain homologues dichotomically grouped with falcipains 1 and 2 (Fig. 3). *Plasmodium falciparum* and its close relative, *P*. *reichenowi*, have three members in the Hb-specific group, namely, falcipains 2A, 2B and 3 (Fig. 3). The chromosomal region where falcipains 2A, 2B and 3 are located is designated a food-vacuole falcipain (*fvf*) locus due to their localization within the parasite (Fig. 4). In *P. falciparum*, the *fvf* locus is located on chromosome 11, with falcipain 2 and falcipain 3 flanked by two conserved genes, histone methyltransferase Set 7 and one of the AP2 transcription factors. Every *Plasmodium* sp. with available genomic sequence shares a similar syntenic pattern with different numbers of falcipain genes at the *fvf* locus (Fig. 4).

*Plasmodium falciparum* has two copies of falcipain 2, falcipain 2A and falcipain 2B that are almost identical (93.4 % identity at the amino acid sequence level) (Fig. 4). The presence of highly similar falcipain 2A and falcipain 2B genes at the same locus can result in gene conversion and promotion of genetic exchanges between the two repertoires, which might be a strong evolutionary driver for improving gene diversity [30] (Additional file 3: Figure S8). *Plasmodium vivax* and *P. knowlesi* also have three falcipain genes at their respective *fvf* locus, but the degree of similarity between the two species is approximately 70 %. There is only one falcipain gene at the *fvf* locus of *P. berghei* and *P. yoelii*, but the gene is still flanked by the conserved histone methyltransferase Set 7 and one of the AP2 transcription factor genes.

Phylogenetic analysis established that members of the falcipain family exist as a common ancestor of *Plasmodium* and independently undergo expansion. There was a diversification between falcipains specific to Hb digestion (falcipains 2 and 3 at the *fvf* locus) and falcipain 1 prior to malarial speciation (Fig. 3). Every *Plasmodium* sp. has a single falcipain 1 gene, and in the primate malaria parasite branch (*P. falciparum*, *P. reichenowi*, *P. vivax*, *P. knowlesi*), Hb-specific falcipain genes have expanded into three copies. *Plasmodium vivax*/*P. knowlesi* branch is clustered together with three matching pairs between the two species, suggesting that the expansion into three genes occurred prior to their speciation. Diversification into falcipain 2 and falcipain 3 in the *P. falciparum*/*P. reichenowi* branch is likely to be an independent event from that of *P. vivax*/*P. knowlesi* branch based on the phylogenetic tree structure (Fig. 3) [29].

It is worth noting that *Theileria parva* and *Theileria annulata*, members of the Apicomplexan species, have a large expansion of their falcipain family with ten putative members in their respective family prior to speciation (Additional file 3: Figure S1). The expansion might have facilitated their growth in both white and red blood cells. Nevertheless, the expansion into ten genes is not a necessity for parasitism in red blood cells since *Plasmodium* and *Babesia* spp. have only three to four falcipain genes.

### Falcilysin family

Falcilysin is a member of metalloprotease family and is localized at the apicoplast and food vacuole [31, 32]. Peptides corresponded to *P. falciparum* falcilysin were identified by mass spectrometry during HDP complex purification [11]. A homologue of falcilysin is found in every *Plasmodium* sp., and the phylogenetic tree demonstrates conventional species evolution in malaria parasites (Additional file 3: Figure S2) [33]. *Plasmodium* falcilysins are highly conserved with more than 70 % amino acid sequence similarity among *Plasmodium* species. Both *Theileria parva* and *Theileria annulata* have two copies of falcilysin, and phylogenetic tree structure indicates that falcilysin duplication occurred before *Theileria* speciation (Additional file 3: Figure S2).

### HDP family

HDP is localized in malaria parasite food vacuole where it detoxifies haem released from digested Hb by facilitating the crystallization of haem into haemozoin [12]. HDP phylogenetic tree structure is similar to that of falcilysin (Additional file 3: Figure S3). *Plasmodium* HDPs are highly conserved and are essential for *P. falciparum* and *P. berghei* intra-erythrocytic development [10]. Blood parasites that do not produce haemozoin, such as *Babesia* and *Theileria*, have only one copy of *P. falciparum* HDP homologue, suggesting that HDP in these two species may have another function or that a different form of haemozoin exists.

### Selective pressure on Hb processing genes

In order to understand the effects of selective pressure of drugs, such as artemisinin and chloroquine, the ratio between single nucleotide polymorphism (SNPs) causing non-synonymous mutations and synonymous mutations (N/S ratio) and the positions of mutated residues in the three-dimensional structures were determined [34, 35]. Bias towards non-synonymous mutations means that the gene of interest is under pressure to diversify, probably due to drug selective pressure. In addition, functional significance of these mutations could be reflected in their locations either at functional motif or as a cluster.

N/S ratios were analysed in samples collected from different parts of the world and those specific to Southeast Asia, a hot spot of anti-malarial resistance. As high-throughput sequencing data for falcipain 2A and 2B are limited by their sequence similarity, nested-PCR falcipain 2A and 2B amplicons were sequenced from artemisinin-resistant ANL2 and ANL4 and artemisinin-sensitive ANL1 and ANL3 strains obtained from the same area [36]. Mutations data are listed in Additional file 4 and Additional file 5.

**Fig. 5:**
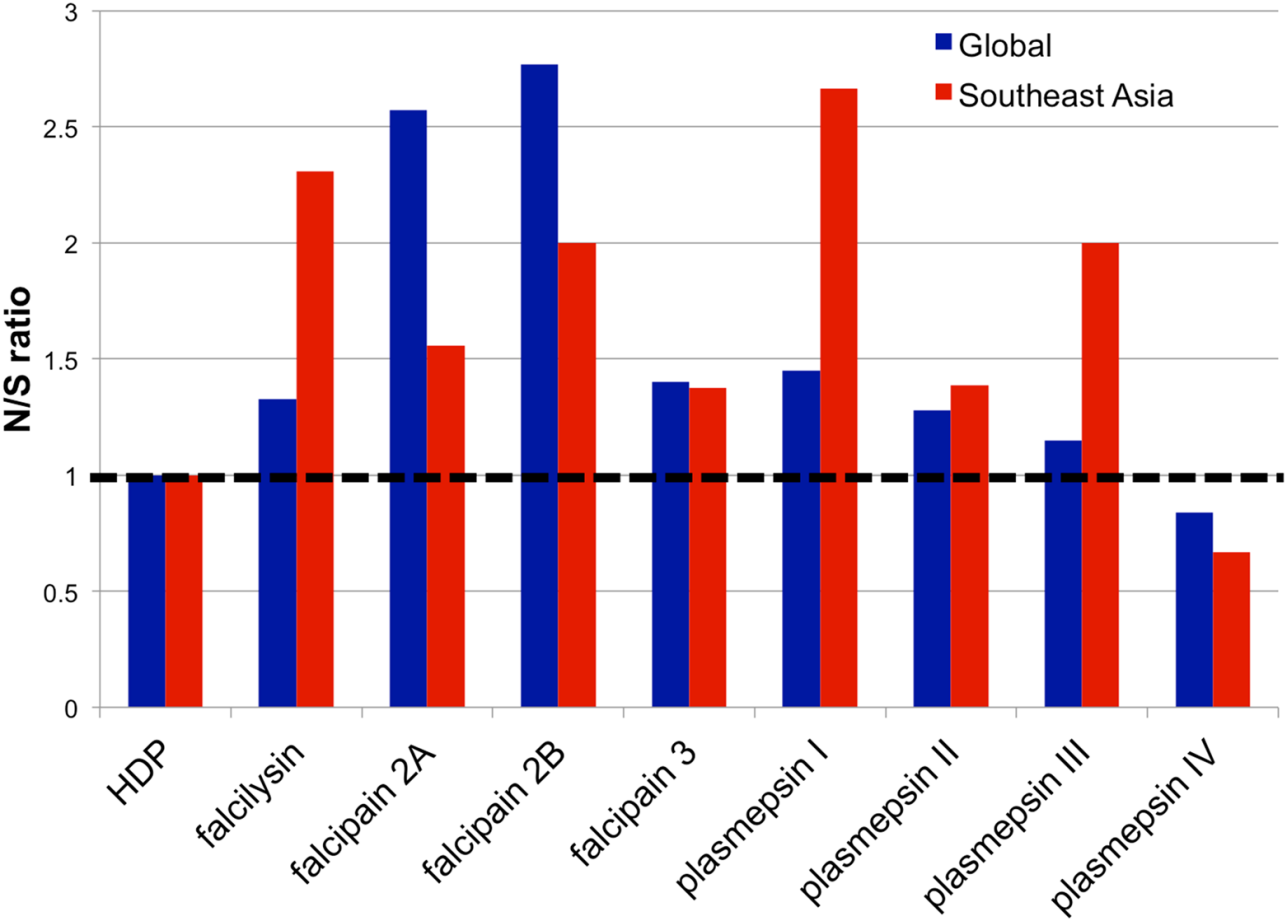
Ratio of non-synonymous and synonymous mutation (N/S ratio) of HDP complex enzymes. Reported mutations from MalariaGen Database were used to calculate N/S ratio. For falcipain 2A and falcipain 2B, mutations found from manual sequencing of ANL1-ANL4 clones were included in the dataset. Ratio above 1 (marked by *dashed line*) might imply positive selection

Analysis of N/S ratio revealed that HDP and the majority of non-Hb targeting plasmepsin genes are not under positive selection (Fig. 5). Worldwide, falcipain 2A and falcipain 2B significantly have more SNPs with non-synonymous than those with synonymous mutations. When the analysis is limited to Southeast Asia, the N/S ratio for falcilysin, falcipain 2B, plasmepsin I, and plasmepsin III are elevated, but, surprising, not that of plasmepsin IV.

### Plasmepsin

**Fig. 6:**
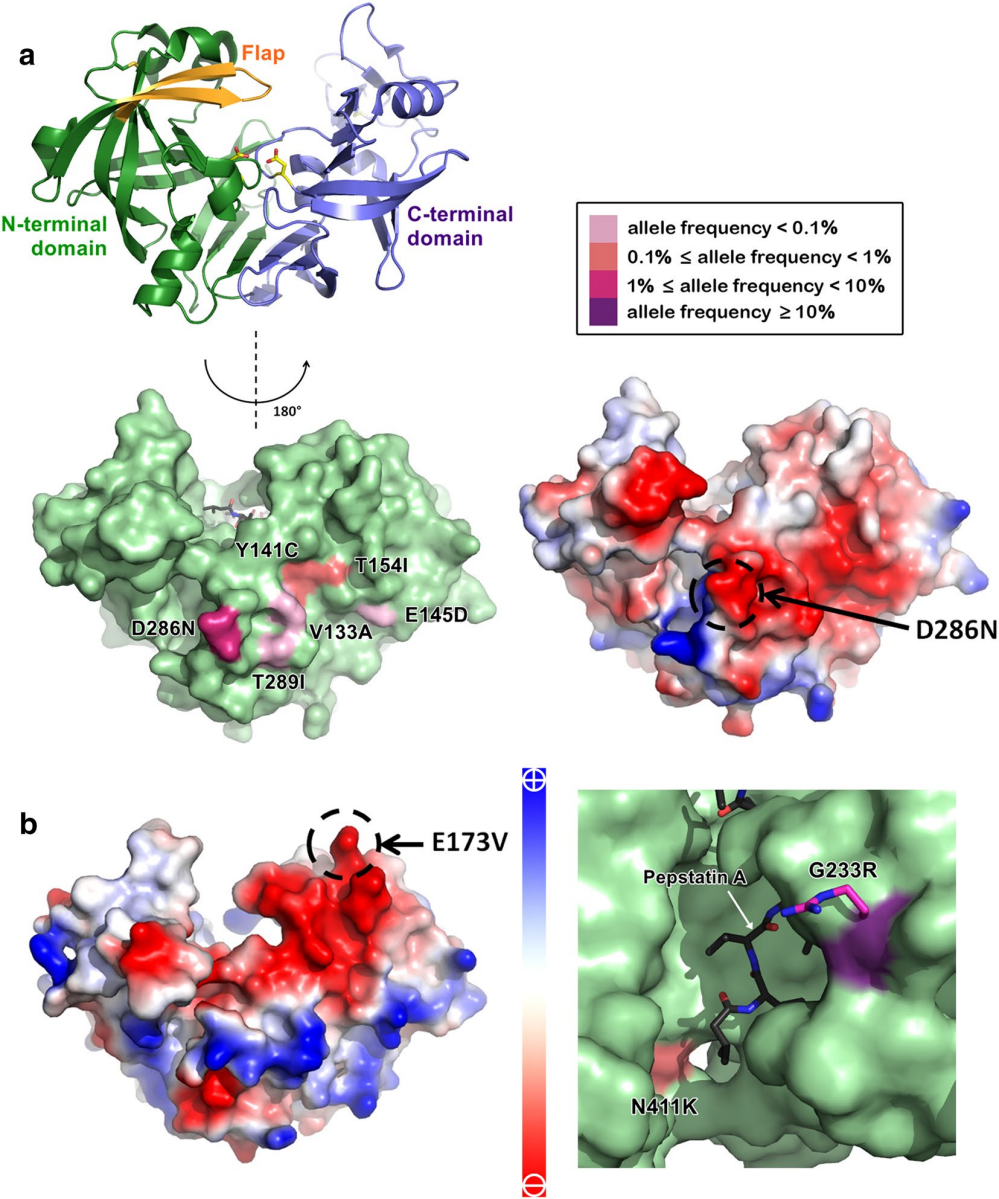
Non-synonymous mutations in plasmepsin II and plasmepsin III. **a** Structural composition of plasmepsin II is shown as a *ribbon diagram* (*top left panel*). The N-terminal domain is shown in *green* and the C-terminal domain is shown in *blue*. The inter-domain β-sheet is located at the bottom of the structure, containing three β-strands from N-terminal and C-terminal domains. The orange β-hairpin forms the flap covering the active site. The catalytic dyad, Asp157 from the N-terminal domain and Asp337 from the C-terminal domain, are coloured in *yellow*. Plasmepsin II mutations are shown on the surface (*bottom left panel*) with colour intensity in *pink shade* representing allele frequency in Southeast Asia. Six mutations are clustered on the surface of plasmepsin II; D286N (1.4 %), Y141C and T154I (0.1 %), V133A, E145D and T289I (<0.1 %). Y141C was found exclusively in Southeast Asia. V133A, E145D, D286N, and T289I are from Southeast Asia and Africa. T154I was found in South Asia, Southeast Asia and Africa. Electrostatic potential surface of plasmepsin II reveals the large negative-charged patch (*bottom right panel*). The surface area contributed by D286 is marked by *dashed circle*. **b** Electrostatic potential surface of plasmepsin III (HAP) reveals large negative-charged patch (*left panel*). The surface area contributed by E173 is identified by *dashed circle*. Mutation E173V could be found with allele frequency <0.1 % in Southeast Asia and 0.1 % in West Africa. Mutation N411K (*right panel, pink surface*), which is a residue lining the substrate-binding cleft, is present only in Southeast Asia with the allele frequency of 0.1 %. The G233R mutation (*right panel, purple surface*) on the surface near substrate-binding cleft has allele frequency >20 % in Southeast Asia, South Asia and Papua New Guinea. Arginine at the position 233 was modelled and shown as *purple stick*. Pepstatin A is shown as *black stick*. Plasmepsin II structure was taken from PDB ID: 1XDH. HAP structure was taken from PDB ID: 3FNT

Plasmepsin I-IV have the pepsin-like structure. The overall structure, as in other eukaryotic aspartic proteases, can be divided into N-terminal and C-terminal domains, each composed of highly twisted β-sheets, small α-helices and an intra-domain disulfide bond, with the two domains connected by a six-stranded β-sheet (Fig. 6a) [37]. The N-terminal domain contains a ‘flap’ β-hairpin that covers the active site in the presence of substrate. The active sites of plasmepsin I, II and IV consist of the conventional aspartic protease catalytic dyad that contains two aspartate residues, one from the N- and the other from C-terminal domain. Plasmepsin III (histo-aspartic protease—HAP) catalytic dyad is composed of one histidine and one aspartate residue [38].

The three mutations of plasmepsin I found in Southeast Asia are located in close proximity on the enzyme surface (Additional file 3: Figure S4). N148I and L180H are exclusive to Southeast Asia and I213V is found in Southeast Asia and South Asia. Mutation F112L is also specific to Southeast Asia (allele frequency = 0.2 %) and South Asia (allele frequency = 3.9 %). Its location is in the pro-domain region, which was not included in the structure.

Plasmepsin II mutations (V133A, Y141C, E145D, T154I, D286N, T289I), found in Asia and Africa except for Southeast Asia-specific C141 variant, also form a cluster on the negatively charged surface (Fig. 6a). N286 variant is found more often in Southeast Asia (allele frequency = 1.4 %) than in Africa (allele frequency < 0.1 %). Mutations L321F and A323T/V, found in Asia and Africa, are located on the same side of a β-strand in the interior hydrophobic milieu of the enzyme (Additional file 3: Figure S5). The Southeast Asia-specific L411V mutation is located also within the enzyme interior and close to the substrate-binding cleft (Additional file 3: Figure S5). Mutations N271S and Y302H, located on the surface, are exclusive to Southeast Asia (Additional file 3: Figure S5).

Plasmepsin III mutations N411K and G233R are located near the substrate-binding cleft (Fig. 6b), with K411 variant found only in Southeast Asia (allele frequency = 0.1 %) and R233 variant throughout Asia (allele frequency >20 %). Plasmepsin III has a surface negative-charge patch similar to that of plasmepsin II but at a different location and contains mutation E173V that is not specific to Southeast Asia (Fig. 6b).

Plasmepsin IV mutation P397S, located near the substrate-binding pocket, is specific to Southeast Asia (allele frequency = 0.5 %) and is in the same binding pocket as plasmepsin III K411 variant (Additional file 3: Figure S6). Mutation N268K is equivalent to plasmepsin II N271S, but is not unique to Southeast Asia (Additional file 3: Figure S6). Of the three mutations, N272I, R353T and N268K, N272I variant is specific to Southeast Asia (allele frequency = 0.1 %) and R353T variant is global (allele frequency = 30.8 %).

### Falcipain

**Fig. 7:**
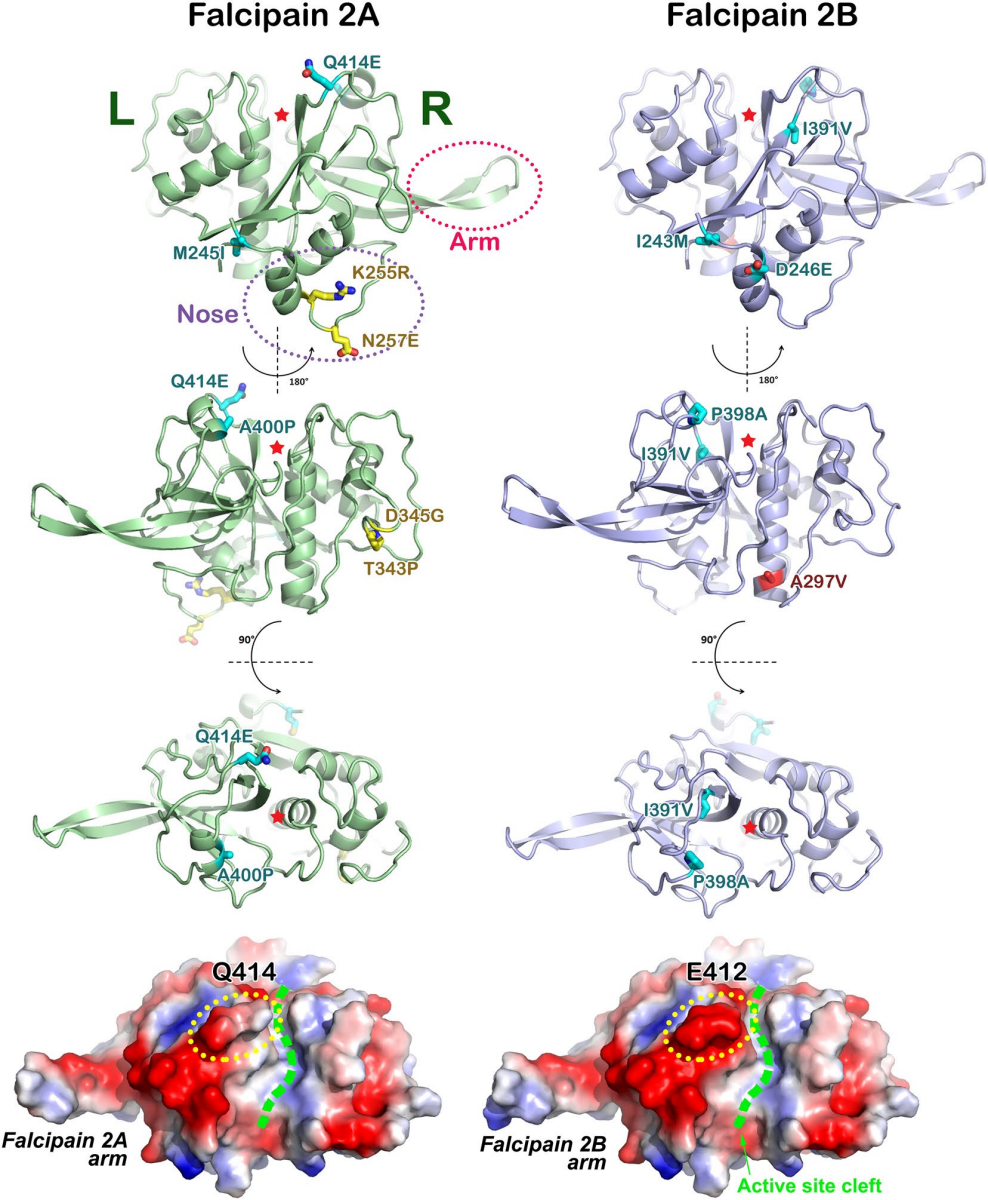
Mutations in falcipain 2A and falcipain 2B. Key domains of falcipain are shown in the *top left panel*. Arm and nose domains are unique in *Plasmodium* falcipain. *Cyan stick* indicates conversion mutation. *Yellow stick* indicates non-conversion mutation that is similar to falcipain 2A variation in W2 strain. *Red stick* indicates non-conversion mutation that does not originate from W2. *Red stars* label the location of active site. Electrostatic potential surface of falcipain 2A and falcipain 2B is shown (*bottom panel*). Surface potential contribution from Q414 and E412 is circled in *yellow dotted line*. *Green dashed lines* mark the active site cleft. Falcipain 2A structure, which was taken from PDB ID: 1YVB is originally from *P. falciparum* strain W2. Falcipain 2B is a homology model using 1YVB as a template

The structures of falcipain 2A and falcipain 3 catalytic domain are similar to other papain-like cysteine proteases (Fig. 7) [39, 40], being composed of a triad C285, H417 and N447 in falcipain 2A and C293, H425 and N455 in falcipain 3. In general, falcipain is structurally composed of five α-helices and nine β-strands, divided into two domains, L (composed mainly of α-helices) and R (containing one β-sheet and two small α-helices), which are arranged sequentially in primary sequence (Fig. 7) [39, 40]. The active site of falcipain is located between the L and R domains. Falcipains contain a unique ‘nose’ domain which is a 16-residue N-terminal extension bridging the L and R domains, and an ‘arm’ domain consisting of β6 and β7 strands from the R domain. The arm region is a putative haemoglobin binding domain (Fig. 7). Deletion of the 10 amino acid residues located at the tip reduced overall haemoglobinase activity without any deleterious effect on protease [41]. So far, no mutation was found at the arm domain.

Falcipain 2A mutation A400P is located in α5 helix (thereby causing a kink) that is close to the substrate-binding cleft and is likely to come from falcipain 2B (Fig. 7). This mutation is found in Asia and Africa (global allele frequency = 0.3 %). Falcipain 2B also has the matching mutation of P398A, again as the result of gene conversion. Falcipain 2B A398 variant is found worldwide (allele frequency = 11 %) but with a lower prevalence (6.4 %) in Southeast Asia.

Falcipain 2A mutation M245I, equivalent to mutation I243M in falcipain 2B, is more prevalent in Africa (allele frequency = 2.1 %) than in Southeast Asia (allele frequency = 0.1 %), but conversely, falcipain 2B M243 variant is more prevalent in Southeast Asia (allele frequency = 2.9 %) than in Africa (allele frequency = 0.6 %). These mutations are located in α1 helix of the ‘nose’ domain (Fig. 7).

Falcipain 2A mutation Q414E, found in *P*. *falciparum* ANL1 and ANL2 strains, is located in the negative-charge patch that extends from the Hb-binding ‘arm’ domain to the surface area near the active site cleft (Fig. 7). In comparison to 3D7, falcipain 2A mutations K255R, N257E, T343P and D345G were found in ANL strains (Additional file 4), and are identical to those in the W2 strain that has a shared origin from Southeast Asia. R255 and E257 substitutions are located close to each other at the ‘nose’ domain, while P343 and G345 substitutions are located in close proximity in a loop connecting α4 helix and β1 sheet at the opposite side of the ‘arm’ domain (Fig. 7).

Falcipain 2B mutations I243M and D246E are present in the ‘nose’ domain, with the former located in α1 helix. Mutation A297V, found in ANL4 strain, is located in α2 helix. I391V mutation, found in ANL1, is located in the interior hydrophobic milieu of the enzyme and has a global allele frequency of 10.5 %.

Falcipain 3 mutations A264V, N371K, R411K, and N468Y were found in Southeast Asia but they are also present in Africa. K371 substitution is located at the negative-charge surface of L domain; K411 and Y468 lie close to the ‘arm’ domain; and V264 is located in the ‘nose’ domain (Additional file 3: Figure S7).

### Falcilysin

**Fig. 8:**
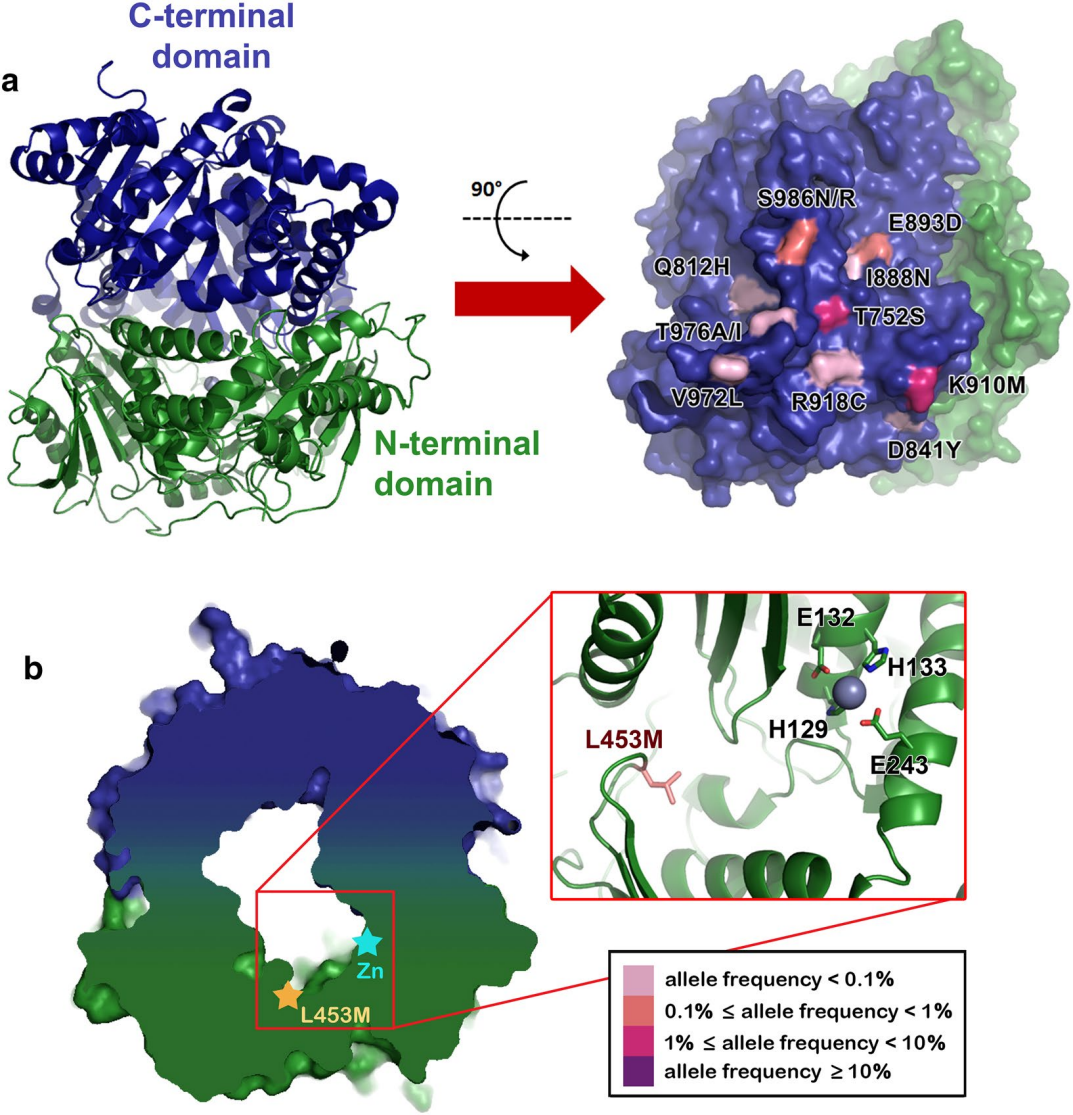
Falcilysin mutations found in Southeast Asia. **a** *Structure of falcilysin* is shown as *ribbon diagram* (*left panel*). The N-terminal and C-terminal domains are coloured in *dark green and blue*, respectively. Clusters of ten Southeast Asia mutations on the outer surface of the C-terminal domain of falcilysin are shown in *pink with shade* representing allele frequency (*right panel*). K910M and S986N are Southeast Asia-exclusive mutations (allele frequencies of 1 and 0.1 %, respectively). S986R could be found predominantly in South Asia (1.4 %), Southeast Asia (0.1 %) and Africa (<0.1 %). T752S and E893D are more common in Southeast Asia (allele frequency of 2.2 and 0.3 %, respectively) but could also be found in Africa (allele frequency 0.1 and <0.1 %, respectively). Q812H, D841Y, I888N, R918C, V972L, and T976A/I are found at low frequencies (<0.1 %) in Southeast Asia and Africa. **b** *Cross-section of falcilysin* showing central cavity. The locations of catalytic zinc atom (*cyan star*) and Southeast Asia-specific L453M (*orange star*) inside the central cavity are indicated. It is the only mutation found in Southeast Asia that is located inside the central cavity. This allele has allele frequency of 0.1 %. Falcilysin active site is shown in the inset. L453M is shown as *pink stick*. Zinc is coordinated by His129, His133 and Glu243. Falcilysin structures were obtained from PDB ID: 3S5K and 3S5I

Falcilysin is a zinc-metalloprotease structurally divided into two similar N-terminal and C-terminal domains, each composed of two αβ rolls, with the catalytic site situated in the central cavity of the first αβ roll of the N-terminal domain (Fig. 8). The zinc-binding motif is composed of residues HXXEHX_109_E and the zinc atom is coordinated to H129, H133 and E243 and a water molecule. It has different substrate specificity depending on pH [42].

Ten mutations (T752S, Q812H, D841Y, I888N, E893D, K910M, R918C, V972L, T976A/I, S986N/R) found in *P. falciparum* worldwide are clustered on the same outer surface of the C-terminal domain (Fig. 8a). M910 (allele frequency = 1 %) and N986 (allele frequency = 0.1 %) variants are Southeast Asia-specific. In addition, mutation L453M, located on the inner surface of the enzyme substrate cavity, is specific to Southeast Asia (allele frequency = 0.1 %) (Fig. 8b).

### HDP

At present, the structural information on HDP is not available. A biochemical study showed the importance of key conserved histidine residues (Histidine 122, 172, 175, and 197 in *P. falciparum*) in haemozoin formation [43]. Four SNPs are found in Southeast Asia at the gene encoding HDP, but only charge-reversion E112K is specific to Southeast Asia (allele frequency = 0.1 %). The data from MalariaGEN suggested that C41F (allele frequency = 0.2 %) was found only in Southeast Asia, but this mutation was previously reported in the HB3 strain originated from South America [44]. The other two mutations, V164I and I185T, can be found in Southeast Asia and Africa.

## Discussion

Changes in gene dosage by gene duplication play an important role in the survival of malaria parasites under drug-induced stress. One of the original observations was the amplification of *P*. *falciparummdr1*, linked to resistance to mefloquine [45]. Amplification of GTP cyclohydrolase I gene, encoding the upstream rate-limiting enzyme in the malaria parasite folate pathway, not only results in antifolate resistance, but also drives the evolution of drug resistance of other genes in the folate pathway [46, 47]. Nevertheless, too many copies of a particular gene could be deleterious to an organism. Mutually exclusive expression of multigene families is often adopted in *P*. *falciparum* to select a sub-set of genes to be expressed at a particular time or stage of development in order to gain a selective advantage [48].

Analysis of Hb processing genes in *Plasmodium* and Apicomplexan spp. has revealed expansion of protease genes (encoding plasmepsin, falcipain and falcilysin) specific to malaria parasites. Hb-specific plasmepsin is encoded by a single gene in every *Plasmodium* sp. except for *P. falciparum*/*P. rechenowi* clade where there are four plasmepsin genes, whereas the remaining members of the family (plasmepsin V-X) that do not degrade Hb are conserved in other *Plasmodium* and Apicomplexan spp. The expansion of Hb-specific plasmepsin genes indicates a strong selective advantage in maintaining large amounts of such plasmepsin. However, loss of plasmepsin I–IV by genetic ablation does not create a lethal phenotype [49]. In addition to the effect of gene dosage on protein expression level, gene expansion promotes genetic diversity by allowing tolerance to deleterious mutations present in one or more of the genes. Plasmepsin gene expansion probably is not due to direct selective pressure of anti-malarial interventions as the phenomenon also is found in *P.reichenowi*. Plasmepsin gene expansion itself is not needed for survival in the human host as *P. vivax* only has one Hb-specific plasmepsin gene copy. It is possible that increased plasmepsin gene expansion promotes Hb consumption in red blood cells allowing effective propagation in Hb-rich mature red blood cells. This notion needs to be tested in other *Plasmodium* spp.

Furthermore, the presence of closely similar genes, e.g., falcipain 2A and 2B, allows genetic variation by means of gene conversion. Falcipain gene duplications at the *fvf* locus are found in both *P*. *falciparum*/*P*. *reichenowi* and *P*. *vivax*/*P*. *knowlesi* clades and have occurred independently based on the constructed phylogenetic tree and close homology between falcipain 2A and 2B.

Two components of the haem processing genes, HDP and falcilysin, have only one copy per genome. It is not unexpected that their deletions are lethal to malaria parasites [10]. The degree of diversification for HDP is low with an N/S ratio of 1 (Fig. 5). Falcilysin does not have a high N/S ratio at the global scale, but this ratio is higher among *P*. *falciparum* isolates in Southeast Asia (Fig. 5).

The majority of non-synonymous mutations in *P. falciparum* falcipain 2A, falcipain 2B and falcipain 3 are located more in the prodomain than in core enzyme, contrary to those observed for plasmepsins (Additional file 5). This preference may indicate a strong requirement for intact enzymes over protein regulation mediated by the prodomain sequence. Even though no direct protease inhibitor has been adopted for malaria treatment, compounds in both 4-aminoquinoline and artemisinin families are known to perturb haemoglobin degradation and haemozoin formation [11]. Their activities could impose selective pressure on haemoglobin-specific proteases since genetic alterations at the Hb processing genes were experimentally linked to reduced drug sensitivity [10, 13].

Selective pressure on Hb processing genes can be observed by analysing the N/S ratio of existing SNPs. N/S ratio analysis revealed that HDP and most non Hb-targeting plasmepsin genes are not under positive selection. On a global scale, falcipain 2A and falcipain 2B significantly have more SNPs with non-synonymous mutations than those with synonymous mutations. When the analysis is limited to parasites from Southeast Asia, N/S ratio for falcilysin, plasmepsin I and plasmepsin III are elevated significantly. Another important factor is gene conversion between falcipain 2A and falcipain 2B. Sequence analysis of a few strains showed extensive exchanges between the two genes. This issue has been largely overlooked because these two genes are considered a ‘blind spot’ in whole genome sequencing. Analysis of these genes by an alternative approach, such as PCR-based sequencing as conducted in this study, may reveal correlations between falcipain mutations and drug resistance, especially given the fact that gene conversion increases genetic diversity [30].

## Conclusion

This study demonstrates that *Plasmodium* Hb processing genes are currently under-appreciated despite the degree of evolutionary pressure to expand and diversify. This is due largely in part to the lack of lethal intra-erythrocytic-stage phenotypes as ex vivo gene knock-out experiments were performed in rich media culture, which may have prevented the generation of such phenotypes. Adaptation of more stringent growth conditions could reveal hidden phenotypes of intra-erythrocytic-stage parasites (Bunditvorapoom et al., unpublished data). Systemic analysis of malaria parasite Hb processing genes will be important in dissecting their roles in developing drug resistance and in pathogenesis.

## Additional files

10.1186/s12936-016-1097-9 List of haemoglobin processing genes.
10.1186/s12936-016-1097-9 Primers used for amplification of *P. falciparum* falcipain 2A and falcipain 2B.
10.1186/s12936-016-1097-9 Additional figure S1–S8.
10.1186/s12936-016-1097-9 List of mutations found in ANL clones.
10.1186/s12936-016-1097-9 MalariaGEN allele frequencies of mutations found in Southeast Asia.
