# Supplementary material for: Comparative genome-wide analysis and evolutionary history of haemoglobin-processing and haem detoxification enzymes in malarial parasites
Source: Malar J. 2016 Jan 29;15:51. doi: 10.1186/s12936-016-1097-9 (PMC4731938; doi:10.1186/s12936-016-1097-9)
Supplement: Supplementary file 1 — 10.1186/s12936-016-1097-9 List of haemoglobin processing genes. [file 12936_2016_1097_MOESM1_ESM.docx]

**Additional file 1** List of haemoglobin processing genes. Name, accession number, and defined code name in phylogenetic trees are described

A. Falcipain group

| Protein name | Accession | Code name |
| --- | --- | --- |
| *Plasmodium falciparum* strain 3D7 | | |
| Falcipain 1 | XP_001348727 | Pf fal1 |
| Falcipain 2A | XP_001347836 | Pf fal2A |
| Falcipain 2B | XP_001347832 | Pf fal2B |
| Falcipain 3 | XP_001347833 | Pf fal3 |
| *Plasmodium reichenowi* strain CDC | | |
| cysteine proteinase falcipain 1 | CD067044 | Pr fal1 |
| cysteine proteinase falcipain 2a | Provided by Otto T. | Pr fal2A |
| cysteine proteinase falcipain 2b | Provided by Otto T. | Pr fal2B |
| cysteine proteinase falcipain 3 | CD064958 | Pr fal3 |
| *Plasmodium vivax* strain Sal-1 | | |
| cysteine proteinase precursor (vivapain-1) | XP_001615807 | Pv fal1 |
| falcipain-like protein (vivapain-4) | XP_001615272 | Pv fal2 |
| falcipain-like protein (vivapain-3) | XP_001615273 | Pv fal3 |
| falcipain-like protein (vivapain-2) | XP_001615274 | Pv fal4 |
| *Plasmodium knowlesi* strain H | | |
| trophozoite cysteine proteinase precursor | XP_002260291 | Pk fal1 |
| falcipain-like protein | XP_002259151 | Pk fal2 |
| falcipain-like protein | XP_002259152 | Pk fal3 |
| falcipain-like protein | XP_002259153 | Pk fal4 |
| *Plasmodium berghei* strain ANKA | | |
| trophozoite cysteine proteinase precursor | XP_677643 | Pb fal1 |
| falcipain 2 precursor, partial | XP_680416 | Pb fal2 |
| *Plasmodium yoelii* strain 17XNL | | |
| hypothetical protein | XP_729023 | Py fal1 |
| falcipain-like protein | XP_726900 | Py fal2 |
| *Babesia bovis* strain T2Bo | | |
| cysteine protease 2 | XP_001610695 | Bb fal1 |
| papain family cysteine protease-containing protein | XP_001612131 | Bb fal2 |
| *Babesia microti* strain RI | | |
| unnamed protein product | CCF76151 | Bm fal1 |
| unnamed protein product | CCF73019 | Bm fal2 |
| unnamed protein product | CCF72975 | Bm fal3 |
| *Eimeria tenella* strain Houghton | | |
| cathepsin B | AEK20867 | Et fal1 |
| putative cathepsin C, partial | AFT64209 | Et fal2 |
| *Toxoplasma gondii* strain ME 49 | | |
| cathepsin L-like thioproteinase, putative | XP_002371694 | Tg fal1 |
| preprocathepsin c precursor, putative | EPT27574 | Tg fal2 |
| cysteine proteinase, putative | XP_002367300 | Tg fal3 |
| papain family cysteine protease domain-containing protein | XP_002368817 | Tg fal4 |
| cathepsin C2 | XP_002371619 | Tg fal5 |
| cathepsin C | XP_002368387 | Tg fal6 |
| *Theileria parva* strain Mugaga | | |
| cysteine proteinase | XP_763301 | Tp fal1 |
| cysteine proteinase | XP_763302 | Tp fal2 |
| cysteine proteinase | XP_763298 | Tp fal3 |
| cysteine protease | XP_764666 | Tp fal4 |
| cysteine proteinase | XP_763303 | Tp fal5 |
| cysteine proteinase | XP_763300 | Tp fal6 |
| cysteine protease | XP_764668 | Tp fal7 |
| cysteine protease | XP_764667 | Tp fal8 |
| cysteine proteinase | XP_763299 | Tp fal9 |
| cysteine proteinase | XP_764709 | Tp fal10 |
| *Theileria annulata* strain Ankara | | |
| cysteine proteinase precursor, tacP | XP_954975 | Ta fal1 |
| cysteine proteinase precursor, tacP | XP_954974 | Ta fal2 |
| cysteine proteinase precursor, tacP | XP_954970 | Ta fal3 |
| cysteine proteinase precursor, tacP | XP_954971 | Ta fal4 |
| cysteine protease precursor, tacP | XP_954973 | Ta fal5 |
| cysteine protease | XP_952610 | Ta fal6 |
| cysteine proteinase precursor, tacP | XP_954976 | Ta fal7 |
| cysteine protease | XP_952609 | Ta fal8 |
| cysteine proteinase precursor, tacP | XP_954972 | Ta fal9 |
| cysteine proteinase | XP_952571 | Ta fal10 |

B. Plasmepsin group

| Protein name | Accession | Code name |
| --- | --- | --- |
| *Plasmodium falciparum* strain 3D7 | | |
| Plasmepsin I | XP_001348249 | Pf PM1 |
| Plasmepsin II | XP_001348250 | Pf PM2 |
| Plasmepsin III | XP_001348251 | Pf PM3 |
| Plasmepsin IV | XP_001348248 | Pf PM4 |
| Plasmepsin V | XP_001349975 | Pf PM5 |
| Plasmepsin VI | XP_001351190 | Pf PM6 |
| Plasmepsin VII | XP_001347613 | Pf PM7 |
| Plasmepsin VIII | XP_001348799 | Pf PM8 |
| Plasmepsin IX | XP_001348455 | Pf PM9 |
| Plasmepsin X | XP_001349441 | Pf PM10 |
| *Plasmodium reichenowi* strain CDC | | |
| plasmepsin I | CDO66563 | Pr PM1 |
| plasmepsin II | CDO66564 | Pr PM2 |
| histo-aspartic protease | CDO66565 | Pr PM3 |
| plasmepsin IV | CDO66562 | Pr PM4 |
| PEXEL protease | CDO66029 | Pr PM5 |
| plasmepsin VI | CDO62543 | Pr PM6 |
| plasmepsin VII | CDO64768 | Pr PM7 |
| plasmepsin VIII | CDO67121 | Pr PM8 |
| plasmepsin IX | CDO66769 | Pr PM9 |
| plasmepsin X | CDO63855 | Pr PM10 |
| *Plasmodium vivax* strain Sal-1 | | |
| aspartic protease | XP_001616871 | Pv PM4 |
| aspartic protease | XP_001615633 | Pv PM5 |
| aspartyl proteinase | XP_001613135 | Pv PM6 |
| aspartyl protease | XP_001608432 | Pv PM7 |
| aspartyl proteinase | XP_001615731 | Pv PM8 |
| aspartyl protease | XP_001616672 | Pv PM9 |
| aspartyl protease | XP_001613486 | Pv PM10 |
| *Plasmodium knowlesi* strain H | | |
| plasmepsin | XP_002260915 | Pk PM4 |
| aspartyl (acid) protease | XP_002260106 | Pk PM5 |
| aspartyl protease | XP_002258928 | Pk PM6 |
| aspartyl protease | XP_002261857 | Pk PM7 |
| aspartyl protease | XP_002260209 | Pk PM8 |
| aspartyl protease | XP_002260721 | Pk PM9 |
| eukaryotic aspartyl protease | XP_002257596 | Pk PM10 |
| *Plasmodium berghei* strain ANKA | | |
| plasmepsin | XP_678821 | Pb PM4 |
| aspartyl (acid) protease | XP_677725 | Pb PM5 |
| plasmepsin VI | CDS45013 | Pb PM6 |
| plasmepsin VII, putative | CDS45303 | Pb PM7 |
| plasmepsin VIII | CDS50465 | Pb PM8 |
| aspartyl protease | XP_680314 | Pb PM9 |
| pepsinogen | XP_677648 | Pb PM10 |
| *Plasmodium yoelii* strain 17XNL | | |
| plasmepsin | XP_727654 | Py PM4 |
| aspartyl protease | XP_729942 | Py PM5 |
| aspartyl protease | CDZ11024 | Py PM6 |
| plasmepsin VII, putative | CDU16776 | Py PM7 |
| plasmepsin VIII, putative | CDU19958 | Py PM8 |
| pepsinogen A | XP_728996 | Py PM9 |
| aspartyl protease | XP_727390 | Py PM10 |
| *Babesia bovis* strain T2Bo | | |
| aspartyl protease | XP_001610711 | Bb PM9 |
| eukaryotic aspartyl protease family protein | XP_001611483 | Bb PM6 |
| aspartyl protease | XP_001610888 | Bb PM7 |
| aspartyl protease | XP_001610957 | Bb PM10 |
| *Babesia microti* strain RI | | |
| unnamed protein product | CCF75661 | Bm PM9 |
| unnamed protein product | CCF75252 | Bm PM5 |
| unnamed protein product | CCF72955 | Bm PM6 |
| unnamed protein product | CCF74973 | Bm PM7 |
| unnamed protein product | CCF74385 | Bm PM10 |
| *Eimeria tenella* strain Houghton | | |
| hypothetical protein, conserved | CDJ41377 | Et PM5 |
| aspartyl proteinase (eimepsin) | CAC20153 | Et PM6 |
| eukaryotic aspartyl protease, putative | CDJ43222 | Et PM9a |
| unknown | ABQ41431 | Et PM9b |
| *Toxoplasma gondii* strain ME 49 | | |
| eukaryotic aspartyl protease, putative | XP_002367480 | Tg ASP |
| aspartyl protease ASP5 | EPT30417 | Tg PM5 |
| eukaryotic aspartyl protease, putative | XP_002365394 | Tg PM6 |
| eukaryotic aspartyl protease, putative | XP_002365884 | Tg PM7 |
| eukaryotic aspartyl protease, putative | XP_002369711 | Tg PM8 |
| eukaryotic aspartyl protease, putative | XP_002367043 | Tg PM9 |
| *Trypanosoma cruzi* strain CL Brener | | |
| No hits | | |
| *Trypanosoma brucei* strain TREU927 | | |
| No hits | | |
| *Theileria parva* strain Mugaga | | |
| cathepsin E | XP_763075 | Tp PM6 |
| pepsinogen | XP_766213 | Tp PM10 |
| aspatyl protease | XP_762800 | Tp PM5 |
| pepsin A | XP_763324 | Tp PM9 |
| *Theileria annulata* strain Ankara | | |
| aspartyl protease precursor | XP_954772 | Tp PM6 |
| pepsinogen | XP_954996 | Ta PM9 |
| pepsinogen | XP_954092 | Ta PM10 |
| aspartyl protease | XP_955372 | Ta PM5 |
| aspartyl protease | XP_953975 | Ta PM7 |

C. Falcilysin group

| Protein name | Accession | Code name |
| --- | --- | --- |
| *Plasmodium falciparum* strain 3D7 | | |
| falcilysin | XP_001350319 | Pf FLN |
| *Plasmodium reichenowi* strain CDC | | |
| falcilysin | CDO66388 | Pr FLN |
| *Plasmodium vivax* strain Sal-1 | | |
| falcilysin | XP_001616391 | Pv FLN |
| *Plasmodium knowlesi* strain H | | |
| falcilysin | XP_002261282 | Pk FLN |
| *Plasmodium berghei* strain ANKA | | |
| bergheilysin | CDS48794 | Pb FLN |
| *Plasmodium yoelii* strain 17XNL | | |
| falcilysin | XP_727812 | Py FLN |
| *Babesia bovis* strain T2Bo | |  |
| Peptidase M16 inactive domain containing protein | XP_001610012 | Bb FLN |
| *Babesia microti* strain RI | | |
| unnamed protein product | CCF76120 | Bm FLN |
| *Eimeria tenella* strain Houghton | | |
| Presequence protease, related | CDJ44497 | Et FLN |
| *Toxoplasma gondii* strain ME 49 | | |
| Peptidase M16 inactive domain-containing protein | EPT27265 | Tg FLN |
| *Trypanosoma cruzi* strain CL Brener | | |
| Pitrilysin-like metalloprotease | XP_817917 | Tc FLN |
| *Trypanosoma brucei* strain TREU927 | | |
| Pitrilysin-like metalloprotease | XP_847019 | Tb FLN |
| *Theileria parva* strain Mugaga | | |
| falcilysin | XP_764826 | Tp FLN1 |
| falcilysin | XP_764823 | Tp FLN2 |
| *Theileria annulata* strain Ankara | | |
| falcilysin | XP_952458 | Ta FLN1 |
| Falcilysin-related protein | XP_952460 | Ta FLN2 |

D. HDP group

| Protein name | Accession | Code name |
| --- | --- | --- |
| *Plasmodium falciparum* strain 3D7 | | |
| HDP | XP_001348620 | Pf HDP |
| *Plasmodium reichenowi* strain CDC | | |
| HDP | CDO66932 | Pr HDP |
| *Plasmodium vivax* strain Sal-1 | | |
| hypothetical protein | XP_001615924 | Pv HDP |
| *Plasmodium knowlesi* strain H | | |
| Fasciclin domain containing protein, conserved | XP_002260406 | Pk HDP |
| *Plasmodium berghei* strain ANKA | | |
| hypothetical protein | XP_677246 | Pb HDP |
| *Plasmodium yoelii* strain 17XNL | | |
| HDP, putative | CDZ17197 | Py HdP |
| *Babesia bovis* strain T2Bo | | |
| hypothetical protein | XP_001612097 | Bb HDP |
| *Babesia microti* strain RI | | |
| unnamed protein product | CCF72775 | Bm HDP |
| *Eimeria tenella* strain Houghton | | |
| hypothetical protein, conserved | CDJ44922 | Et HDP |
| *Toxoplasma gondii* strain ME 49 | | |
| hypothetical protein | XP_002369186 | Tg HDP |
| *Trypanosoma cruzi* strain CL Brener | | |
| No hits | | |
| *Trypanosoma brucei* strain TREU927 | | |
| No hits | | |
| *Theileria parva* strain Mugaga | | |
| hypothetical protein | XP_764528 | Tp HDP |
| *Theileria annulata* strain Ankara | | |
| hypothetical protein | XP_953512 | Ta HDP |
