# Supplementary material for: Comparative genome-wide analysis and evolutionary history of haemoglobin-processing and haem detoxification enzymes in malarial parasites
Source: Malar J. 2016 Jan 29;15:51. doi: 10.1186/s12936-016-1097-9 (PMC4731938; doi:10.1186/s12936-016-1097-9)
Supplement: Supplementary file 2 — 10.1186/s12936-016-1097-9 Primers used for amplification of P. falciparum falcipain 2A and falcipain 2B. [file 12936_2016_1097_MOESM2_ESM.docx]

**Additional file 2** Primers used for amplification of *P. falciparum* falcipain 2A and falcipain 2B.

| Fragment | Primer name | Primer sequence (5′ to 3′) | Length (nt) | Product size (bp) |
| --- | --- | --- | --- | --- |
| Long PCR for Falcipain 2a | | | | |
| LongFal2a | LongFal2a-F | GTGTATTTTATTTTGTAGCAAGAACG | 26 | 1807 |
|  | LongFal2a-R | GTTATAAAATTCTTTCTATCCTTTGGAG | 28 |  |
| Nested PCR for Falcipain 2a | | | | |
| NestFal2a-1 | LongFal2a-F | GTGTATTTTATTTTGTAGCAAGAACG | 26 | 485 |
|  | NestFal2a-1R | CTTGCCATTAGGGCTTTTTAGC | 22 |  |
| NestFal2a-2 | NestFal2a-2F | CTCTTCAGTTGAAAATAATAATG | 23 | 513 |
|  | NestFal2a-2R | GAATTCTTTAATGGTTTTGAAG | 22 |  |
| NestFal2a-3 | NestFal2a-3F | CAGATTTGCCGATTTAACTTATCATG | 26 | 528 |
|  | NestFal2a-3R | GGTCCCAAGAATCTAAGTGCTTC | 23 |  |
| NestFal2a-4 | NestFal2a-4F | GATAGATGTACTGAAAAATATGGAATC | 27 | 512 |
|  | LongFal2a-R | GTTATAAAATTCTTTCTATCCTTTGGAG | 28 |  |
| Long PCR for Falcipain 2b | | | | |
| LongFal2b | LongFal2b-F | GAACTATAAATTTGTGTAAAGG | 22 | 1768 |
|  | LongFal2b-R | GAAAAAATATACATGTTAATACATACAAC | 29 |  |
| Nested PCR for Falcipain 2b | | | | |
| NestFal2b-1 | LongFal2b-F | GAACTATAAATTTGTGTAAAGG | 22 | 413 |
|  | NestFal2a-1R | CTTGCCATTAGGGCTTTTTAGC | 22 |  |
| NestFal2b-2 | NestFal2a-2F | CTCTTCAGTTGAAAATAATAATG | 23 | 490 |
|  | NestFal2b-2R | CGAAGATCTTAAAGTAAGATATTTAC | 26 |  |
| NestFal2b-3 | NestFal2b-3F | TAGATTTGCTGATTTAACTTATCAC | 25 | 528 |
|  | NestFal2a-3R | GGTCCCAAGAATCTAAGTGCTTC | 23 |  |
| NestFal2b-4 | NestFal2a-4F | GATAGATGTACTGAAAAATATGGAATC | 27 | 431 |
|  | LongFal2b-R | GAAAAAATATACATGTTAATACATACAAC | 29 |  |
