# Supplementary material for: Comparative genome-wide analysis and evolutionary history of haemoglobin-processing and haem detoxification enzymes in malarial parasites
Source: Malar J. 2016 Jan 29;15:51. doi: 10.1186/s12936-016-1097-9 (PMC4731938; doi:10.1186/s12936-016-1097-9)
Supplement: Supplementary file 3 — 10.1186/s12936-016-1097-9 Additional figure S1–S8. [file 12936_2016_1097_MOESM3_ESM.docx]

**Additional file 3** Additional Figure S1-S8.


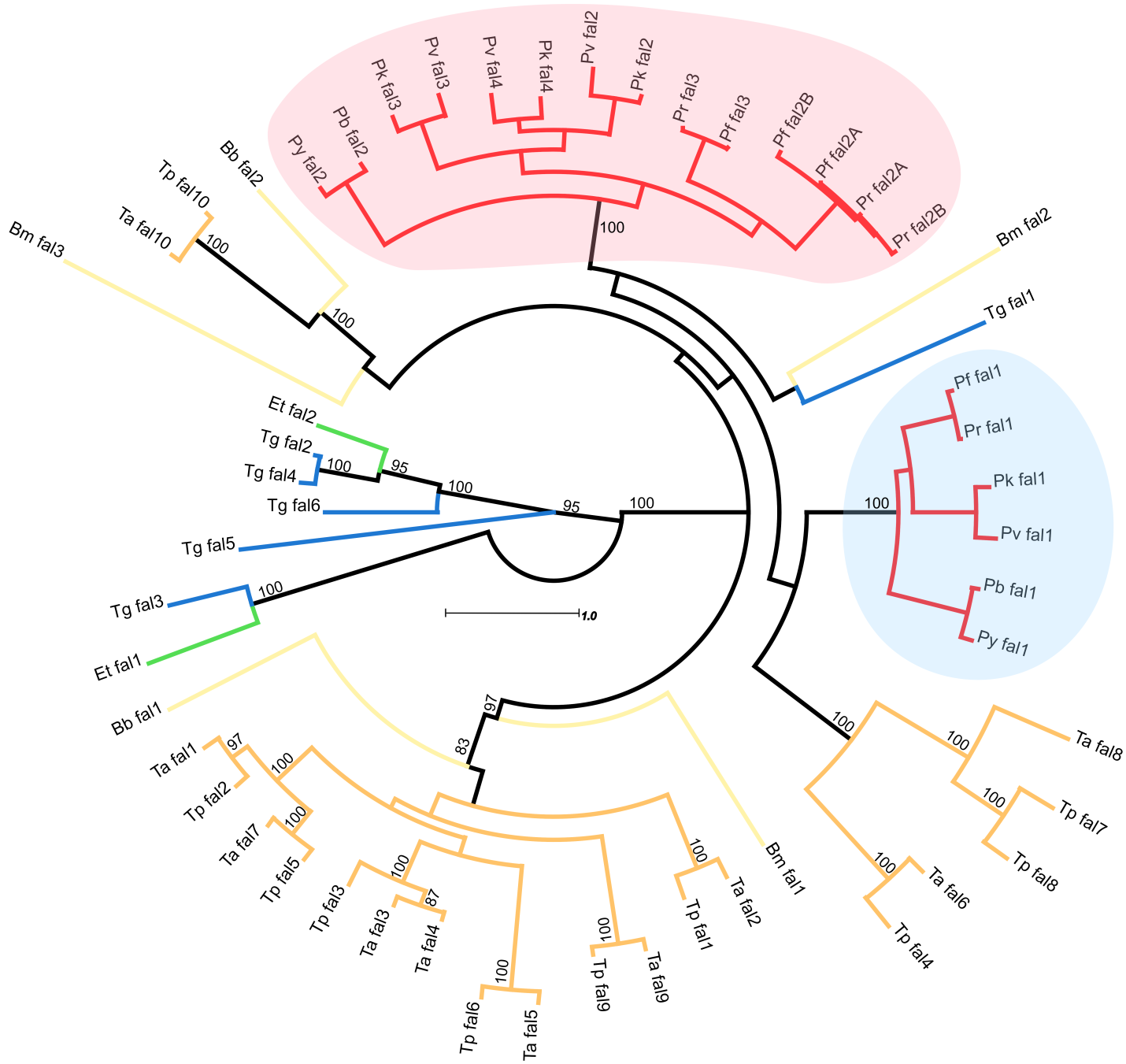


**Figure S1** ML phylogenetic tree of falcipain. Separation of invasion-specific falcipain (light blue area) and haemoglobin-specific falcipain (pink area) is evident in *Plasmodium* (red branch). Expansion of *P. falciparum* falcipain homologs occurred in *Theileria* (orange branch) and *Trypanosoma* (purple branch). Sequences shown here were given code name (see Additional file 1). ML support value above 75% based on 100 replicates bootstrap is shown on the branch except *Plasmodium* clade (red branch). Star indicates that the node has <75% bootstrap support based on 1000 replicates for Neighbor-Joining tree. Red branch: Pf, *P. falciparum*; Pr, *P. reichenowi*; Pv, *P. vivax*; Pk, *P. knowlesi*; Pb, *P. berghei*; Py, *P. yoelii*; Yellow branch: Bb, *B. bovis*; Bm, *B. microti*; Orange branch: Tp, *T. parva*; Ta, *T. annulata*; Green branch: Et, *E. tenella*; Blue branch: Tg, *T. gondii*.


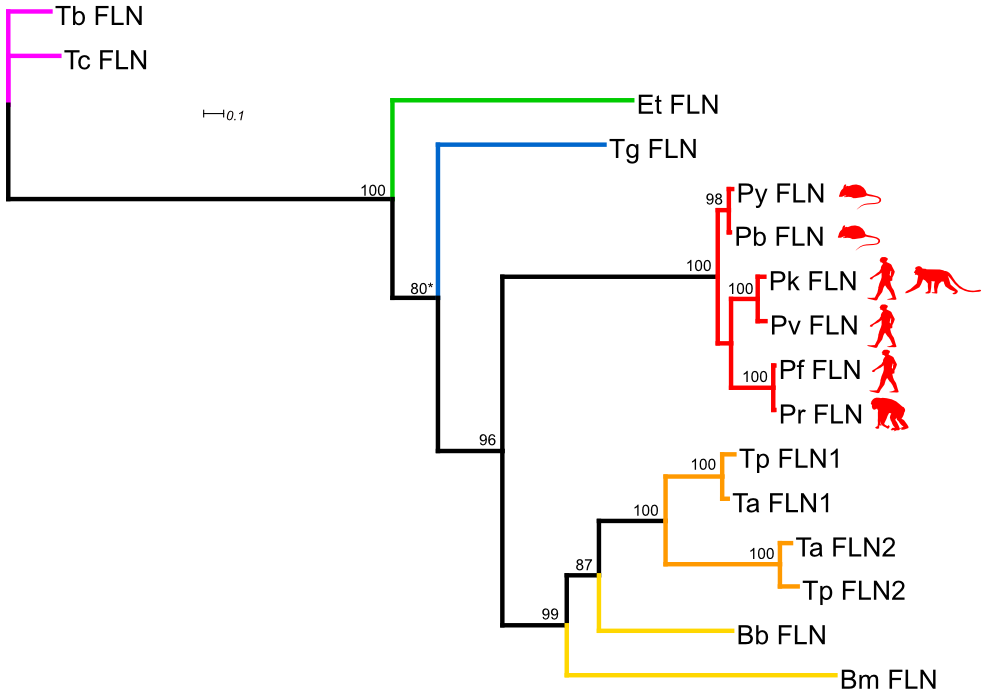


**Figure S2** ML phylogenetic tree of falcilysin. Expansion of *P. falciparum* falcilysin homologues occurred in *Theileria* (orange branch). ML support value above 75% based on 100 replicates bootstrap is shown on the branch. Star indicates that the node has <75% bootstrap support based on 1000 replicates for Neighbor-Joining tree. Red branch: Pf, *P. falciparum*; Pr, *P. reichenowi*; Pv, *P. vivax*; Pk, *P. knowlesi*; Pb, *P. berghei*; Py, *P. yoelii*; Yellow branch: Bb, *B. bovis*; Bm, *B. microti*; Orange branch: Tp, *T. parva*; Ta, *T. annulata*; Green branch: Et, *E. tenella*; Blue branch: Tg, *T. gondii*; Purple branch: Tb, *T. brucei*; Tc, *T. cruzi*. Sequences shown here were given code name (see Additional file 1)


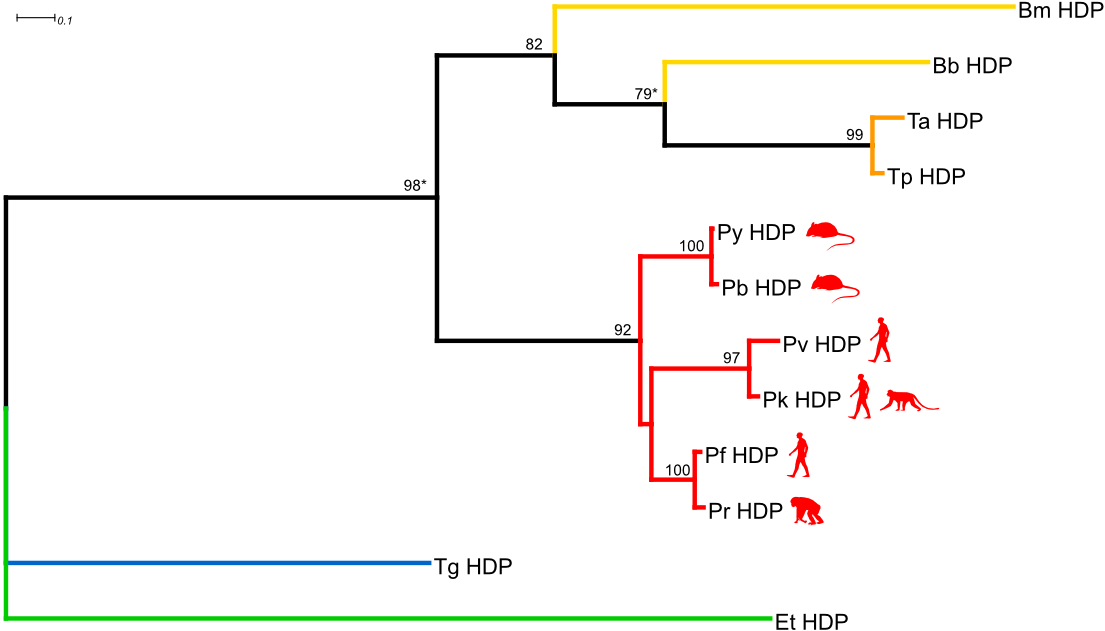


**Figure S3** ML phylogenetic tree of HDP. ML support value above 75% based on 100 replicates bootstrap is shown on the branch. Star indicates that the node has <75% bootstrap support based on 1000 replicates for Neighbor-Joining tree. Red branch: Pf, *P. falciparum*; Pr, *P. reichenowi*; Pv, *P. vivax*; Pk, *P. knowlesi*; Pb, *P. berghei*; Py, *P. yoelii*; Yellow branch: Bb, *B. bovis*; Bm, *B. microti*; Orange branch: Tp, *T. parva*; Ta, *T. annulata*; Green branch: Et, *E. tenella*; Blue branch: Tg, *T. gondii*. Sequences shown here were given code name (see Additional file 1)


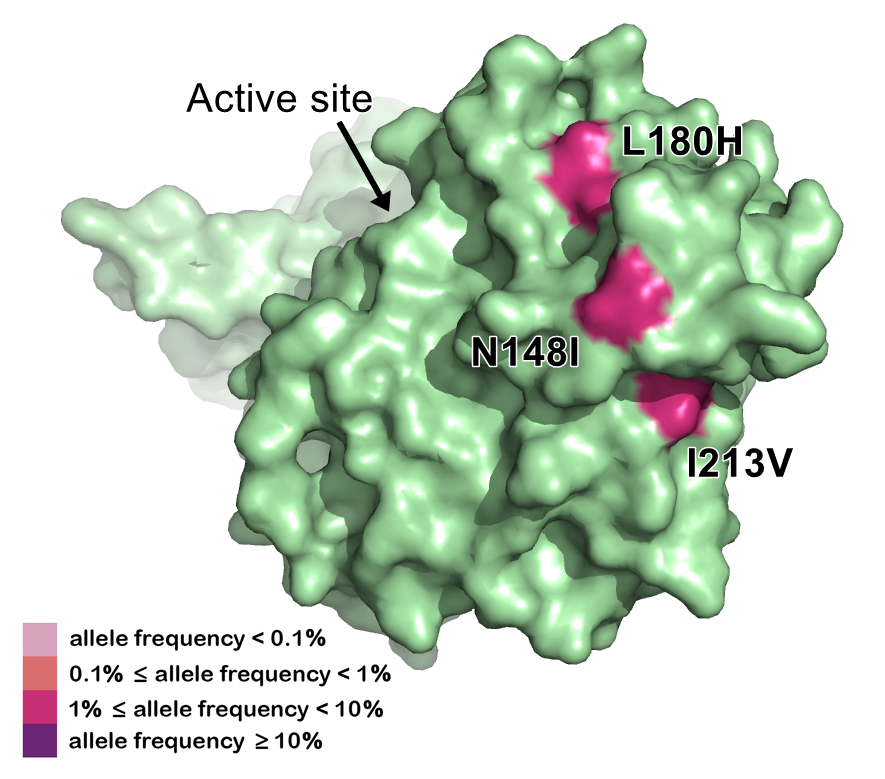


**Figure S4** Non-synonymous mutations in plasmepsin I. N148I (1.7% allele frequency), L180H (3.3% allele frequency) and I213V (1.8% allele frequency) are located on the same side on the surface of the protein. Two of them (L180H and N148I) were found exclusively in Southeast Asia. The other, I213V, was found only in Southeast and South Asia. Plasmepsin I structure was taken from PDB ID: 3QSI.


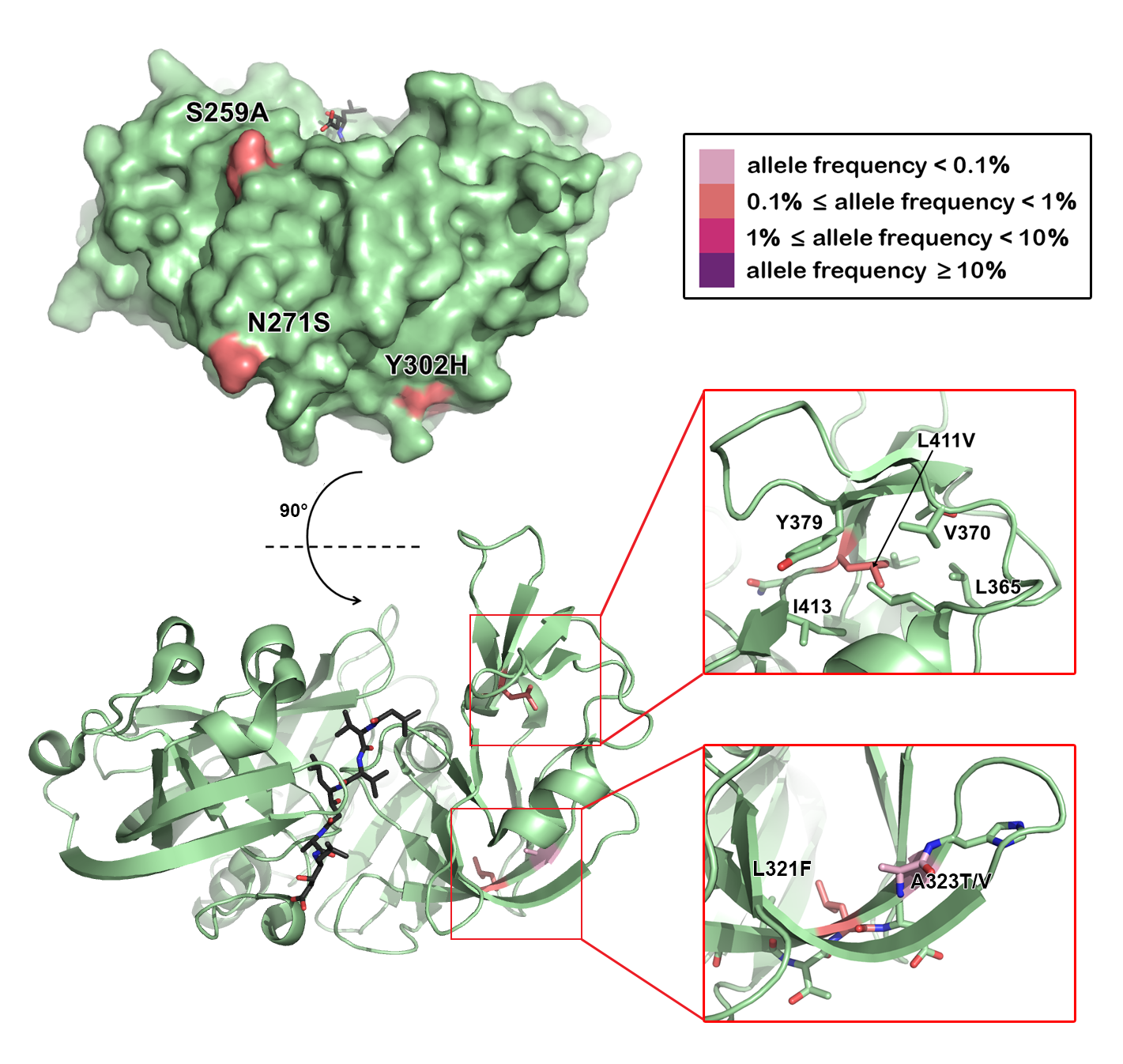


**Figure S5** Non-synonymous mutations in plasmepsin II. Three plasmepsin II mutations are found scattered on the opposite site from the mutation cluster shown in Figure 6A. Two of them, N271S (allele frequency 0.1%) and Y302H (allele frequency 0.1%), are found exclusively in Southeast Asia. Another mutation, S259A, is found in Southeast Asia (allele frequency 0.5%) and in East Africa. Three mutations of plasmepsin II are buried in the core. L321F (allele frequency 0.3%) and A323T/V (allele frequency <0.1% each) were found in Southeast Asia and Africa. Another mutation, L411V, was found exclusively in Southeast Asia with the allele frequency of 0.2%. The inhibitor (pepstatin A) is shown as a black stick. Plasmepsin II structure was taken from PDB ID: 1XDH.


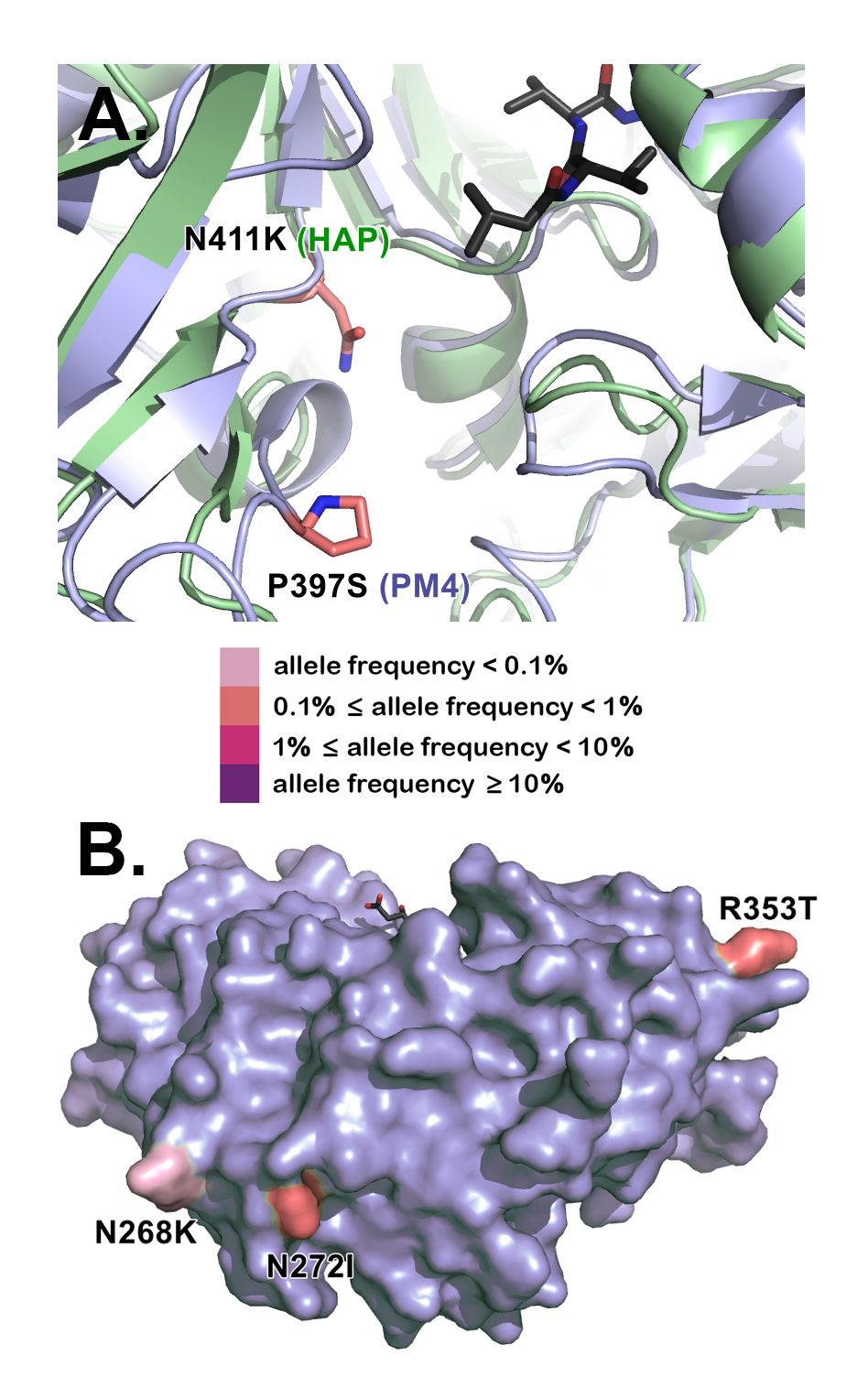


**Figure S6** Non-synonymous mutations in plasmepsin III (HAP) and plasmepsin IV. (A) Superimposition of plasmepsin IV (light blue) and plasmepsin III (pale green) shows mutations that occupy the same space near the active site. Both mutations were found specifically in Southeast Asia. Plasmepsin IV P397S has allele frequency of 0.5%. Plasmepsin III N411K has allele frequency 0.1%. (B) Plasmepsin IV mutations found on the surface are shown. N272I is found exclusively in Southeast Asia with the allele frequency 0.1%. R353T has allele frequency of 0.6% in Southeast Asia but has higher allele frequency elsewhere (allele frequency 18-52%, see Additional file 5). N268K has allele frequency <0.1%, but it is homologous to N271S of plasmepsin II. N268K is found in Southeast Asia and Africa. Pepstatin A from plasmepsin III structure is shown as the black stick. Plasmepsin III structure was taken from PDB ID: 3FNT. Plasmepsin IV structure was taken from PDB ID: 1LS5.


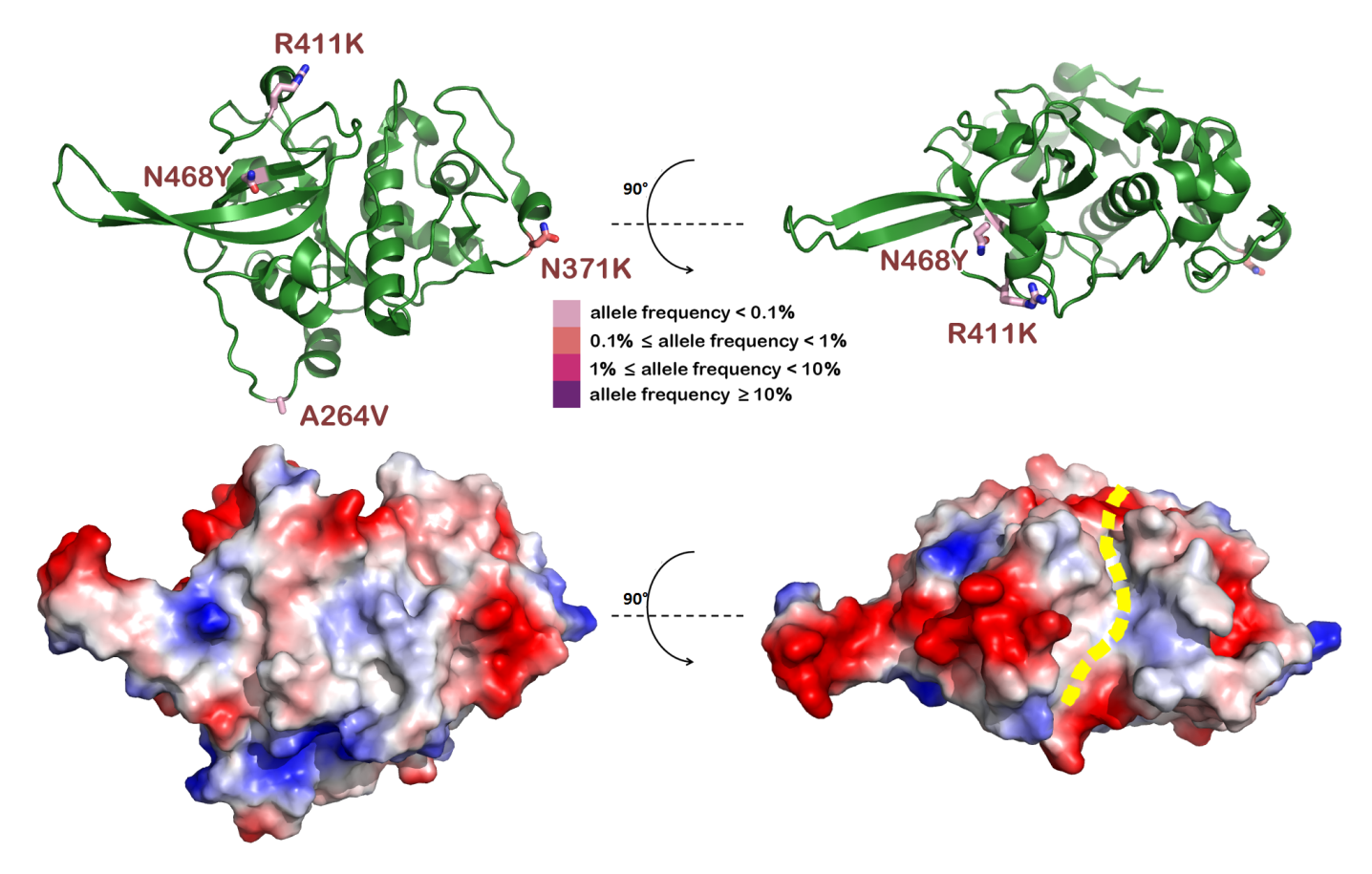


**Figure S7** Falcipain 3 mutations found in Southeast Asia. A264V is found at allele frequency <0.1% in Southeast Asia and Africa. N468Y and R411K are prevalent in Africa but are also found at low frequency (<0.1%) in Southeast Asia. N371K is found in Southeast Asia (allele frequency 0.1%) and South Asia (allele frequency 3.5%) but it could also be found at higher frequencies in Africa and South America (allele frequency 22-37%). Electrostatic potential surface of falcipain 3 (bottom panel) shows the negative patch extending from the arm to the active site (yellow dash line). Falcipain 3 structure was taken from PDB ID: 3BPM.


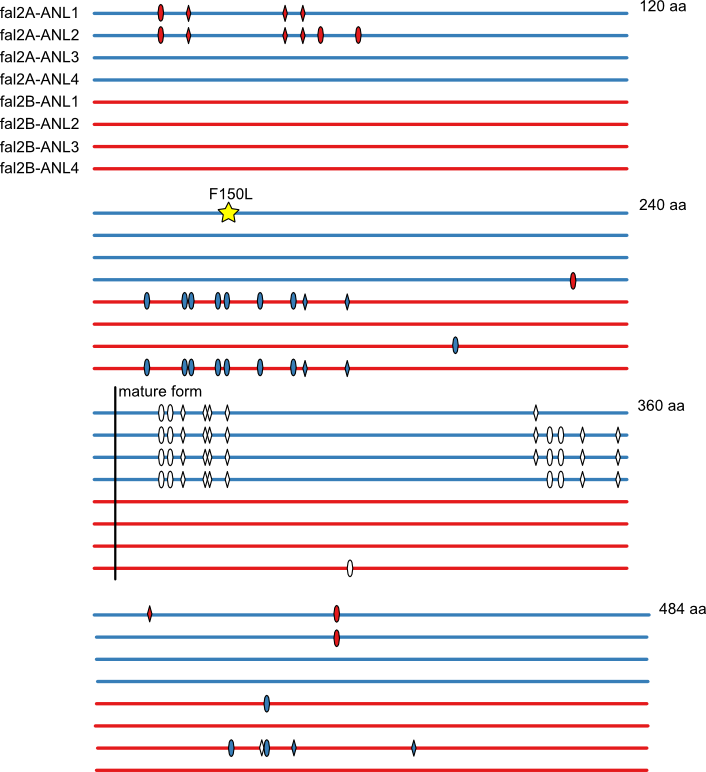


**Figure S8** Gene conversion in falcipain 2A (blue line) and falcipain 2B (red line) found in ANL parasites. Red symbols are mutations in falcipain 2A that comes from falcipain 2B with oval as non-synonymous mutations and diamond as synonymous mutations. The blue symbols are based on the same pattern but represent the conversion from falcipain 2A to falcipain 2B. White symbols are mutations that are not likely to be the result of gene conversion, but are identical to *P. falciparum* W2, a standard strain from Southeast Asia. F150L (yellow star) is the only non-conversion mutation which does not match with that of W2. Mature form of falcipain 2A starts at residue 244.
