# Supplementary material for: Comparative genome-wide analysis and evolutionary history of haemoglobin-processing and haem detoxification enzymes in malarial parasites
Source: Malar J. 2016 Jan 29;15:51. doi: 10.1186/s12936-016-1097-9 (PMC4731938; doi:10.1186/s12936-016-1097-9)
Supplement: Supplementary file 4 — 10.1186/s12936-016-1097-9 List of mutations found in ANL clones. [file 12936_2016_1097_MOESM4_ESM.docx]

**Additional file 4** List of mutations found in ANL clones. Mutation arose from gene conversion between falcipain 2A and falcipain 2B is shaded in blue. Star (*) indicates the mutation that Sanger sequencing disagrees with high-throughput sequencing data from MalariaGen. Mutation which is identical to *P. falciparum* W2 strain is noted (†). Two mutations of ANL2 are heterozygous (**).

| **Mutation** | **ANL-1** | **ANL-2** | **ANL-3** | **ANL-4** |
| --- | --- | --- | --- | --- |
| **Falcipain 2A** | | | | |
| **Q15H** | **✓** | **✓** | ✕ | ✕* |
| **V51I** | ✕ | **✓** | ✕ | ✕ |
| **S59F** | ✕ | **✓** | ✕ | ✕ |
| **F150L** | **✓** | ✕ | ✕ | ✕ |
| **S228T** | ✕ | ✕ | ✕ | **✓** |
| **K255R†** | **✓** | **✓** | **✓** | **✓** |
| **N257E†** | **✓** | **✓** | **✓** | **✓** |
| **T343P†** | ✕ | **✓** | **✓** | **✓** |
| **D345G†** | ✕ | **✓** | **✓** | **✓** |
| **Q414E** | **✓** | **✓** | ✕ | ✕ |
| **Falcipain 2B** | | | | |
| **K132T** | **✓** | ✕ | ✕ | **✓** |
| **E141K** | **✓** | ✕ | ✕ | **✓** |
| **N142D** | **✓** | ✕ | ✕ | **✓** |
| **V147F** | **✓** | ✕ | ✕ | **✓** |
| **H150N** | **✓** | ✕ | ✕ | **✓** |
| **V157A** | **✓** | ✕ | ✕ | **✓** |
| **T165M** | **✓** | ✕ | ✕ | **✓** |
| **K202N** | ✕ | ✕ | **✓** | ✕ |
| **A297V** | ✕ | ✕ | ✕ | **✓** |
| **I391V** | ✕ | ✕ | **✓** | ✕ |
| **P398A** | **✓** | ✕ | **✓** | ✕ |
| **Plasmepsin I** | | | | |
| **L180H** | ✕ | **✓**** | **✓** | ✕ |
| **Plasmepsin III (HAP)** | | | | |
| **G233R** | **✓** | **✓**** | ✕ | **✓** |
| **Falcilysin** | | | | |
| **I10F** | **✓** | **✓** | **✓** | **✓** |
| **HDP** | | | | |
| **F91L** | **✓** | **✓** | **✓** | **✓** |
