# Supplementary material for: Comparative genome-wide analysis and evolutionary history of haemoglobin-processing and haem detoxification enzymes in malarial parasites
Source: Malar J. 2016 Jan 29;15:51. doi: 10.1186/s12936-016-1097-9 (PMC4731938; doi:10.1186/s12936-016-1097-9)
Supplement: Supplementary file 5 — 10.1186/s12936-016-1097-9 MalariaGEN allele frequencies of mutations found in Southeast Asia. [file 12936_2016_1097_MOESM5_ESM.docx]

**Additional file 5** MalariaGEN allele frequencies of mutations found in Southeast Asia. Mutation in bold face indicates that it is exclusive to Southeast Asia. Shade represents allele frequency based on MalariaGen dataset consistent with the scale in Figures.

| **Plasmepsin I** | | | | | |
| --- | --- | --- | --- | --- | --- |
| **Mutation** | **Southeast Asia Mainland (%)** | **Papua New Guinea (%)** | **South Asia (%)** | **Africa (%)** | **South America (%)** |
| A19S | <0.1 | 0 | 0 | 0.4 | 0 |
| T50I | <0.1 | 0 | 0 | <0.1 | 11.1 |
| L57I | <0.1 | 0 | 0 | 0.1 | 0 |
| F112L | 0.2 | 0 | 3.9 | 0 | 0 |
| A125V | <0.1 | 0 | 0 | <0.1 | 0 |
| **N148I** | **1.7** | **0** | **0** | **0** | **0** |
| P166S | 0.2 | 0 | 0 | <0.1 | 0 |
| **L180H** | **3.3** | **0** | **0** | **0** | **0** |
| I213V | 1.8 | 0 | 1.3 | 0 | 0 |
| **V263A** | **0.2** | **0** | **0** | **0** | **0** |
| T333N | <0.1 | 0 | 0 | 0.1 | 0 |
| T347A | <0.1 | 0 | 0 | 1.8 | 0 |
| R383I | <0.1 | 0 | 0 | 0.2 | 0 |
| N387I | <0.1 | 0 | 0 | 0.1 | 0 |
| T442N | <0.1 | 0 | 0 | <0.1 | 0 |
| A448S | 0.1 | 0 | 0 | 0.2 | 0 |
| **Plasmepsin II** | | | | | |
| **Mutation** | **Southeast Asia Mainland (%)** | **Papua New Guinea (%)** | **South Asia (%)** | **Africa (%)** | **South America (%)** |
| N76I | <0.1 | 0 | 0 | <0.1 | 0 |
| E78K | 0.2 | 0 | 1.3 | <0.1 | 0 |
| K95R | <0.1 | 0 | 0 | 0.1 | 0 |
| V133A | <0.1 | 0 | 0 | 0.9 | 0 |
| **Y141C** | **0.1** | **0** | **0** | **0** | **0** |
| E145D | <0.1 | 0 | 0 | 0.1 | 0 |
| T154I | 0.1 | 0 | 1.3 | 0.5 | 0 |
| S259A | 0.5 | 0 | 0 | <0.1 | 0 |
| **N271S** | **0.1** | **0** | **0** | **0** | **0** |
| D286N | 1.4 | 0 | 0 | <0.1 | 0 |
| T289I | <0.1 | 0 | 0 | 0.7 | 0 |
| **Y302H** | **0.1** | **0** | **0** | **0** | **0** |
| L321F | 0.3 | 0 | 0 | 0.1 | 0 |
| A323T | <0.1 | 0 | 0 | <0.1 | 0 |
| A323V | <0.1 | 0 | 0 | 0.1 | 0.1 |
| V370A | <0.1 | 0 | 0 | <0.1 | 0 |
| **L411V** | **0.2** | **0** | **0** | **0** | **0** |
| A449S | <0.1 | 0 | 0 | 0.1 | 0 |
| **Histo-aspartic protease (HAP)-Plasmepsin III** | | | | | |
| **Mutation** | **Southeast Asia Mainland (%)** | **Papua New Guinea (%)** | **South Asia (%)** | **Africa (%)** | **South America (%)** |
| V26I | <0.1 | 0 | 0 | 0.1 | 0 |
| **L49W** | **<0.1** | **0** | **0** | **0** | **0** |
| F63L | 0.1 | <0.1 | 0 | <0.1 | 0 |
| **Q64R** | **<0.1** | **0** | **0** | **0** | **0** |
| K144N | <0.1 | 0 | 0 | 0.1 | 0 |
| E173V | <0.1 | 0 | 0 | 0.1 | 0 |
| **D193Y** | **<0.1** | **0** | **0** | **0** | **0** |
| I216V | <0.1 | 0 | 0 | 0.2 | 0 |
| G233R | 26.9 | 35.8 | 23.9 | 0 | 0 |
| G257D | <0.1 | 10.1 | 0 | <0.1 | 0 |
| Q274K | <0.1 | 0 | 0 | <0.1 | 0 |
| V327I | <0.1 | <0.1 | 0 | 0.1 | 0 |
| Q352K | <0.1 | <0.1 | 0 | 0.1 | 0 |
| L380I | <0.1 | 0 | 0 | 0.1 | 0 |
| **N411K** | **0.1** | **0** | **0** | **0** | **0** |
| I412V | <0.1 | 0 | 0 | 0.1 | 0 |
| **Plasmepsin IV** | | | | | |
| **Mutation** | **Southeast Asia Mainland (%)** | **Papua New Guinea (%)** | **South Asia (%)** | **Africa (%)** | **South America (%)** |
| L57S | 0.3 | 0 | 0 | <0.1 | 0 |
| R65H | <0.1 | 0 | 1.9 | 1.6 | 0 |
| L67F | <0.1 | 0 | 0 | 0.1 | 0 |
| H76Y | 1.6 | 0 | 3.0 | 4.0 | 0 |
| S92R | 0.1 | 1.1 | 0 | 0 | 0 |
| S92N | <0.1 | 0 | 0 | <0.1 | 0 |
| Y138F | <0.1 | 0 | 0 | 0.1 | 0 |
| N268K | <0.1 | 0 | 0 | 0.3 | 0 |
| **N272I** | **0.1** | **0** | **0** | **0** | **0** |
| V281I | <0.1 | 0 | 0 | 0.3 | 0 |
| R353T | 0.6 | 46.6 | 18.2 | 40.8 | 51.9 |
| **P397S** | **0.5** | **0** | **0** | **0** | **0** |
| **Falcipain 2A** | | | | | |
| **Mutation** | **Southeast Asia Mainland (%)** | **Papua New Guinea (%)** | **South Asia (%)** | **Africa (%)** | **South America (%)** |
| A8V | <0.1 | 0 | 0 | 0.9 | 0 |
| Q15H | 76.6 | 86.6 | 83.9 | 8.0 | 18.7 |
| R19I | <0.1 | 0 | 0 | 0 | 3.4 |
| **I110T** | **0.2** | **0** | **0** | **0** | **0** |
| Y113N | <0.1 | 0 | 0 | 0.1 | 0 |
| A122S | 0.3 | 0 | 0 | <0.1 | 0 |
| P135S | <0.1 | 0 | 0 | <0.1 | 0 |
| P135Q | 0.1 | 0 | 0 | <0.1 | 0 |
| K143E | 0.2 | 0 | 1.7 | 0.9 | 0 |
| D144N | 0.2 | <0.1 | 1.7 | 0.9 | 0 |
| F149V | <0.1 | 0 | 1.5 | 0.4 | 0.3 |
| F150L | 2.4 | 0 | 20.0 | 0 | 0 |
| **F154L** | **<0.1** | **0** | **0** | **0** | **0** |
| A159T | 0.2 | 0 | 0 | <0.1 | 0 |
| **E210K** | **0.2** | **0** | **0** | **0** | **0** |
| A215S | 0.1 | 0 | 0 | 0.1 | 0 |
| N224S | 0.3 | 0 | 5.8 | <0.1 | 0 |
| R230I | <0.1 | 0 | 0 | <0.1 | 0 |
| M245I | 0.1 | 0.1 | 0.3 | 2.1 | 0.1 |
| A400P | 0.9 | 0 | 0.1 | <0.1 | 0 |
| **Falcipain 2B** | | | | | |
| **Mutation** | **Southeast Asia Mainland (%)** | **Papua New Guinea (%)** | **South Asia (%)** | **Africa (%)** | **South America (%)** |
| R19G | 0.2 | 0 | 0 | 0.3 | 0 |
| M107K | 2.6 | 2.3 | 0.6 | 0.6 | 0 |
| **D109G** | **0.5** | **0** | **0** | **0** | **0** |
| N113Y | 0.2 | 0 | 0 | 0.1 | 12.5 |
| K132T | 56.7 | 0.8 | 22.2 | 9.8 | 0 |
| **P133S** | **0.2** | **0** | **0** | **0** | **0** |
| E141K | 53.0 | 2.6 | 12.5 | 15.4 | 0 |
| N142D | 50.4 | 2.6 | 12.5 | 14.6 | 0 |
| T165M | 49.6 | 1.3 | 22.4 | 12.3 | 7.4 |
| A213S | <0.1 | 0 | 0 | 0.1 | 0 |
| K221E | <0.1 | 0 | 0 | 0.1 | 0 |
| S222N | 0.1 | 0 | 0 | 0.4 | 0 |
| T226S | 0.2 | 0.2 | 0.2 | 2.5 | 0 |
| I243M | 2.9 | 2.3 | 2.3 | 0.6 | 0 |
| D246E | 0.3 | 0 | 0.6 | 0.2 | 0 |
| I391V | 3.8 | 20.1 | 9.3 | 12.2 | 10.5 |
| P398A | 6.4 | 8.0 | 17.8 | 10.9 | 15.0 |
| **Falcipain 3** | | | | | |
| **Mutation** | **Southeast Asia Mainland (%)** | **Papua New Guinea (%)** | **South Asia (%)** | **Africa (%)** | **South America (%)** |
| N74S | <0.1 | 0 | 0 | 0.3 | 0 |
| A114E | <0.1 | <0.1 | 0 | 0.1 | 0 |
| Q149K | 0.2 | 0 | 0 | 0.1 | 0 |
| M188I | 0.9 | 0 | 0 | <0.1 | 0 |
| R215W | <0.1 | 0 | 0 | 0.1 | 0 |
| G239V | <0.1 | 0 | 0 | 0.1 | 0 |
| V248I | <0.1 | 0 | 0 | 0.1 | 0 |
| A264V | <0.1 | 0 | 0 | <0.1 | 0 |
| N371K | 0.1 | 1.1 | 3.5 | 22.7 | 37.0 |
| R411K | <0.1 | 0 | 0 | 0.5 | 0 |
| N468Y | <0.1 | 0 | 0.4 | 4.6 | 0 |
| **Falcilysin** | | | | | |
| **Mutation** | **Southeast Asia Mainland (%)** | **Papua New Guinea (%)** | **South Asia (%)** | **Africa (%)** | **South America (%)** |
| I10F | 99.3 | 95.7 | 97.0 | 18.7 | 29.6 |
| T49I | 0.1 | 0 | 0 | <0.1 | 0 |
| **Q97R** | **0.1** | **0** | **0** | **0** | **0** |
| **A224D** | **2.2** | **0** | **0** | **0** | **0** |
| **S235T** | **0.2** | **0** | **0** | **0** | **0** |
| D332N | <0.1 | 0 | 0 | 0.1 | 0 |
| E408D | <0.1 | 0 | 0 | 0.1 | 0 |
| **L453M** | **0.1** | **0** | **0** | **0** | **0** |
| M487L | 1.3 | 0 | 0 | <0.1 | 0 |
| M563R | 0.1 | 0 | 3.8 | 0 | 0 |
| E623G | <0.1 | 2.2 | 0 | 0 | 0 |
| P656H | <0.1 | 0 | 0 | <0.1 | 0 |
| T662I | <0.1 | 0 | 0 | 0.1 | 0 |
| D686H | 2.2 | 2.3 | 0 | 0 | 0 |
| D689Y | <0.1 | 0 | 0 | 0.1 | 0 |
| T752S | 2.2 | 0 | 0 | 0.1 | 0 |
| Q812H | <0.1 | 0 | 0 | <0.1 | 0 |
| D828E | <0.1 | 0 | 0 | <0.1 | 0 |
| A833V | <0.1 | 0 | 0 | 0.1 | 0 |
| D841Y | <0.1 | <0.1 | 0 | 0.1 | 0 |
| I888N | <0.1 | 0 | 0 | 0.1 | 0 |
| E893D | 0.3 | 0 | 0 | <0.1 | 0 |
| **K910M** | **1.0** | **0** | **0** | **0** | **0** |
| R918C | <0.1 | 0 | 0 | 0.1 | 0 |
| H942Y | <0.1 | 0 | 0 | 0.1 | 0 |
| V972L | <0.1 | 0 | 0 | 0.1 | 0 |
| T976A | <0.1 | 0 | 0 | 0.1 | 0 |
| T976I | <0.1 | 0 | 0 | 0.1 | 0 |
| **S986N** | **0.1** | **0** | **0** | **0** | **0** |
| S986R | 0.1 | 0 | 1.4 | <0.1 | 0 |
| **Haem detoxification protein (HDP)** | | | | | |
| **Mutation** | **Southeast Asia Mainland (%)** | **Papua New Guinea (%)** | **South Asia (%)** | **Africa (%)** | **South America (%)** |
| **C41F** | **0.2** | **0** | **0** | **0** | **0** |
| **E112K** | **0.1** | **0** | **0** | **0** | **0** |
| V164I | 0.5 | 0 | 0 | 0.1 | 0 |
| I185T | <0.1 | 0 | 0 | 0.1 | 0 |
